# Genome wide transcriptome analysis reveals vital role of heat responsive genes in regulatory mechanisms of lentil (*Lens culinaris* Medikus)

**DOI:** 10.1038/s41598-019-49496-0

**Published:** 2019-09-10

**Authors:** Dharmendra Singh, Chandan Kumar Singh, Jyoti Taunk, Vasudha Jadon, Madan Pal, Kishor Gaikwad

**Affiliations:** 1 Division of Genetics, ICAR-Indian Agricultural Research Institute, New Delhi, 110012 India; 2 Division of Plant Physiology, ICAR-Indian Agricultural Research Institute, New Delhi, 110012 India; 3 ICAR-National Research Centre on Plant Biotechnology, Pusa Campus, New Delhi, 110012 India

**Keywords:** Agricultural genetics, Heat

## Abstract

The present study reports the role of morphological, physiological and reproductive attributes viz. membrane stability index (MSI), osmolytes accumulations, antioxidants activities and pollen germination for heat stress tolerance in contrasting genotypes. Heat stress increased proline and glycine betaine (GPX) contents, induced superoxide dismutase (SOD), ascorbate peroxidase (APX) and glutathione peroxidase (GPX) activities and resulted in higher MSI in PDL-2 (tolerant) compared to JL-3 (sensitive). *In vitro* pollen germination of tolerant genotype was higher than sensitive one under heat stress. *In vivo* stressed pollens of tolerant genotype germinated well on stressed stigma of sensitive genotype, while stressed pollens of sensitive genotype did not germinate on stressed stigma of tolerant genotype. *De novo* transcriptome analysis of both the genotypes showed that number of contigs ranged from 90,267 to 104,424 for all the samples with N_50_ ranging from 1,755 to 1,844 bp under heat stress and control conditions. Based on assembled unigenes, 194,178 high-quality Single Nucleotide Polymorphisms (SNPs), 141,050 microsatellites and 7,388 Insertion-deletions (Indels) were detected. Expression of 10 genes was evaluated using quantitative Real Time Polymerase Chain Reaction (RT-qPCR). Comparison of differentially expressed genes (DEGs) under different combinations of heat stress has led to the identification of candidate DEGs and pathways. Changes in expression of physiological and pollen phenotyping related genes were also reaffirmed through transcriptome data. Cell wall and secondary metabolite pathways are found to be majorly affected under heat stress. The findings need further analysis to determine genetic mechanism involved in heat tolerance of lentil.

## Introduction

Lentil (*Lens culinaris* Medikus) is grown in relatively dry ecology where yield is mainly affected by various abiotic stresses. Increased maximum and minimum growth temperature can result in heat stress in plants. Heat stress can accelerate plant maturity and have detrimental effects on productivity of lentil^1^. Optimum temperature for growth of lentil ranges between 18–30 °C^2^. Temperature ≥30 °C can affect plant growth and development adversely and may lead to yield reduction. Kumari *et al*. showed that seed yield reduced by 38–58 per cent at 33/28 °C^3^. Heat stress may result in scorching and sunburn of leaves, accelerating leaf senescence; inhibition of root and shoot growth and seedling survival^1^. It also reduces membrane stability index (MSI), which reflects leaching of ions due to plasma membrane injury under heat stress. Further, heat stress hinders the photochemical activity of photosystem II (PS-II) which results in imbalance between photosynthesis and respiratory processes. Generation of reactive oxygen species (ROS) due to heat stress leads to oxidative damage of plant cells unless it is scavenged by enzymes such as superoxide dismutases (SOD), catalases (CAT) and peroxidases^4,5^. In addition, osmoprotactant solutes such as proline, glycine betaine and sugars gets accumulated due to activation of various complex regulatory networks^6^. Most of these traits at seedling stage have been strongly correlated with reproductive stage leading to significant losses in seed yield^1,7,8^.

Heat stress induces several signal transduction pathways which are inter connected at cellular level^9,10^. Plant’s survival under heat stress depends on perception, generation and transmission of stress signals along with initiation of required physiological and biochemical responses. Heat tolerance mechanism employs production of compatible solutes for ion transportation, osmo-protection and to maintain cellular structures. At molecular level, heat stress induces expression of genes and production of defence proteins, antioxidants and synthesis of metabolites along with transcriptional controls which are activated to reduce stress alterations^11,12^. Cytosolic protein misfolding acts as important thermo sensor especially during lenient temperature increase where protein injury is certain. Also, fluidity sensitive Ca^2+^ channels in plasma membrane are equally responsible. A transient Ca^2+^ influx was observed in several plants within few minutes of rise in temperature^13^. Specific CAMs and MAP kinases are associated in downstream events transferring the heat signals from membrane to transcription factors^14^. Expression of CaM3 and CaM7 under heat stress has been known in *Arabidopsis*^15,16^. Members of the family of heat shock transcription factors (HSFs) act as transcriptional regulators that plays a vital role in certain heat signalling and are found to be involved in regulation of thousands of downstream targets^17,18^. HSFs activated due to heat stress recognize and bind conserved DNA sequences called heat shock elements in HSP promoters^19^. Activated HSFs displace histones from HSP genes and results in plant acquired thermo-tolerance. Timely expression of HSPs under heat stress is crucial for plants to obtain thermo-tolerance^20^. Cytoskeleton is also involved in heat stress and cause regulation of HSP expression. Thermo tolerance is also regulated by plant hormones such as salicylic acid and abscisic acid^21^.

Information on classification of various stress-responsive genes or gene families is insufficient in non-model legumes like lentil, pigeonpea, mungbean etc. under abiotic stress conditions. Apparently, function of candidate genes in response to drought stress has been identified in lentil^7^. Identification of molecular markers i.e. simple sequence repeats (SSRs) and single nucleotide polymorphisms (SNPs), is a leading target of functional genomic studies. These markers are highly polymorphic, co-dominant, convenient, highly reproducible, stable and authentic. Therefore, identification of such markers in heat stress contrasting genotypes will significantly enrich the recently developed reference genomic pool of lentil i.e. knowpulse.usask.ca.

Illumina platform, a short-read technology has been widely applied to generate large scale genome and transcriptome data in grain legumes such as lentil, chickpea and soybean under various abiotic stress conditions^7,22,23^. However, no published record is available on transcriptome data in response to heat stress in lentil. Therefore, objectives of this study were to (i) identify differentially expressed genes (DEGs) in heat stressed genotypes, (ii) identify role of candidate genes associated with heat tolerance and (iii) identify SSR, SNP and Insertion-deletion (Indel) markers in heat tolerant and sensitive genotypes. This study will provide a new guide for heat stress tolerance at molecular level which paves the way for improvement of commercial lentil genotypes under heat stress utilizing various breeding and biotechnological strategies. Also, newly identified markers can be utilized in molecular and comparative studies in other legumes as well.

## Material and Methods

### Material

Two contrasting genotypes viz. PDL-2 and JL-3, (tolerant and sensitive to heat stress) were selected for genome wide differential expression of transcripts in response to heat stress. PDL-2 is a breeding line (ILL-590 × ILL-7663) received from ICARDA- Syria. JL-3 is a landrace and was obtained from Sagar, Madhya Pradesh, India. Both the genotypes were found to be morpho-physiologically and genetically distinct from each other in response to drought and heat stress conditions in earlier studies^1,24^.

## Methods

### Evaluation of genotypes for heat tolerance under hydroponic condition

Genotypes were evaluated under heat stress using hydroponic conditions as per protocol of Singh *et al*. with little modifications^1^. After surface sterilization using 1% sodium hypochlorite, seeds were germinated on filter paper. Seven days old plants were shifted to nutrient medium having composition as described by Simon *et al*.^25^. Growth chamber was maintained for relative humidity (%), temperature of 55.7/68.2% ± 2, 27/16 °C and 50.6/57.2% ± 2, 35/33 °C during day/night and with a photoperiod of 10/14 h to mimic control and heat stress conditions, respectively^1^. Duration of heat stress was 3 h daily for 3 d, whereas control conditions remained unaltered. Completely randomised design comprising 3 replicates for each genotype and each replicate having 12 seedlings was followed.

### Physiological analysis for heat stress tolerance

Leaves from seven days old seedlings were collected from control and stress treated plants for evaluation of physiological parameters viz. membrane stability index (MSI), glycine betaine content, proline content, antioxidants activities [SOD, peroxidase (POD), glutathione peroxidase (GPX) and ascorbate peroxidase (APX)] and lipid peroxidation using methods as described by Singh *et al*.^7^.

### Analysis of *in vitro* and *in vivo* pollen germination for heat stress tolerance

Pollen germination was done following Singh *et al*.^1^. Hand pollinations were performed to examine pollen germination and tube growth during *in* *vivo* germination. Five flowers were collected after 30 min of incubation and were pollinated to observe germination as well as pollen tube growth on stigma. Each flower was considered as a replication. Each stigma (flower) was pollinated with pollen grains from a particular flower. Flowers were fixed in 80% alcohol for 24 h. Pistils (styles and ovary) were separated, cleared with 6N NaOH for 48 h and gently washed with water. Fluorescent dye aniline blue (0.1%) was used for staining pistils which were observed under fluorescent microscope (Zeiss AXIOSKOP 2)^26^.

Two cross combinations using stressed stigma (35/20 °C) of sensitive genotype (JL-3) x stressed pollen (35/20 °C) of tolerant genotype (PDL-1); non-stressed stigma of tolerant genotype (PDL-1) x stressed pollen (35/20 °C) of sensitive genotype (JL-3) were made to determine site of sensitivity (pollen or stigma) for heat stress. Five flowers were pollinated in each cross combination and each genotype was exposed to 35/20 °C and 27/16 °C (day/night) conditions.

### Ribonucleic acid (RNA) extraction and complementary deoxyribonucleic acid (cDNA) library preparation

Three biological replicates from each of tolerant and sensitive genotypes under control and heat stress conditions were sampled. Each replicate consisted of 12 seedlings which were pooled together (Additional File 1. Fig. S1). Leaf samples were frozen in liquid nitrogen and preserved at −80 °C and were used for RNA extraction for Illumina library construction and quantitative real time polymerase chain reaction (RT-qPCR). TRIZOL reagent (Takara, Japan) was used for extraction of total RNA from control and treated leaf samples followed by RNA integrity test using Bioanalyzer. Library preparation for all the samples was accomplished using TruseqTM RNA sample prep Kit (Illumina, Inc. San Diego, CA, USA). Enriched messenger RNA (mRNA) was sheared into short fragments with the help of magnetic beads containing poly-T molecules. Two hundred base pair (bp) size inserts were selected for post cDNA preparation using Illumina TruSeqTM mRNA Library preparation kit following standard producer’s protocol. Illumina adapters were then ligated to these fragments after their ends were repaired. Clusters were generated and sequencing was done on Illumina HiSeq 2000 platform to generate 2 × 100 bp paired end reads.

### *De novo* assembly of short reads

FastXTool kit was used to clean low quality reads (phred quality below 20). Quality of reads pre and post filtering was analyzed using FASTQC tool. Quality filtered reads were taken for *de novo* assembly using Trinity denovo assembler. *De novo* contigs were produced by assembling clean paired end reads. Unigenes i.e. non-redundant transcripts were obtained from those contigs which could not be further extended on either end following redundancy removal process using sequence clustering software. Bowtie software mapped the clean reads back to the assembled transcripts. Flow chart depicting the bioinformatics analysis of this study is represented in Additional File 2. Fig. S2.

### Annotation and classification of gene function

Transcripts were annotated using Basic Local Alignment Search Tool (BLAST) program (E value < 1.0E^−5^) with the help of National Centre for Biotechnology Information (NCBI) non-redundant (nr), Kyoto Encyclopedia of Genes and Genomes (KEGG), Cluster of Orthologous Groups (COG), Swiss-Protand UniProt Reference Clusters (UNIREF) databases. Gene Ontology (GO) terms for characterization of unigene depicting nr annotation results was accomplished by Annocript program followed by construction of GO tree using Web Gene Ontology Annotation Plot (WEGO) tool. Further, functional classification of unigenes was deduced using COG database. KEGG pathway database was used to predict different pathways for all the unigenes. Representation of DEGs involved in tolerant vs sensitive genotypes under heat stress and control conditions was done using various plots like HeatMap, Volcano Plot and Circos. MapMan (v3.51R2) was used for understanding roles of DEGs in different combination sets.

### SNP, SSR, Indel filtering and calling

SAMtools mpileup and custom scripts were used to identify SNPs and to call variants on the basis of read depth. Read depth of minimum 10, filtered the heterozygous loci as well as false positive SNPs. Genome Analysis Tool Kit (GATK) was used for SNP calling using haplotype caller command version 3.6–0, where all the parameters were set as default. For SSRs, MIcro SAtellite identification (MISA) software deduced SSRs from high quality filtered reads aligned to the contigs. Primer3 software was assisted to design primers where parameters set for designing the primers were 15 bp for minimum, 21 bp for maximum and 18 bp for optimal primer size. Product sizes ranged between 100 to 300 bp from these SSRs.

### Validation of genic SSRs (g-SSRs)

Fifty five g-SSRs were selected and developed to screen 96 genotypes that included cultivars, breeding lines, landraces and wild accessions (Additional File 16. Table S1). These markers were selected on the basis of minimum SSR length of 8mers and GC content < 33.3% (Additional File 17.Table S2). PCR conditions for amplification were similar to Singh *et al*.^1^.

### Validation using RT-qPCR

RNA isolated from the samples using Trizol reagent was used for RT-qPCR. One Step SYBR PrimeScript RT-PCR Kit II (Takara Biotechnology Co. Ltd, Dalian, Japan) was done for validation of 10 gene specific primers though RT-qPCR following the manufacturer’s instructions. Reaction volume (10 µl) contained 100 ng of total RNA normalized for all the samples using CFX96 Touch RT-PCR detection system (Bio-Rad, Hercules). Details of 10 primers used in RT-PCR is presented in Additional File 18. Table S3. To calculate relative expression, 2^−∆∆Ct^ method for target gene expression was used. Beta-tubulin reference gene was taken as internal expression gene to normalize expressed data.

## Results

### Phenotypic responses under heat stress

**Figure 1:**
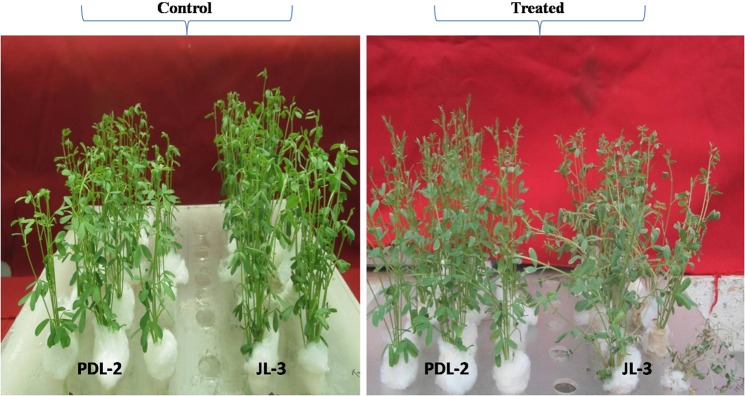
Plant exposed to heat stress (35/33 °C) and control (27/16 °C) in JL-3 (sensitive) and PDL- 2 (tolerant) genotypes at seedling stage in growth chamber under hydroponic conditions.

All plants of both the genotypes were found healthy under optimal 27/16 °C (day/night) condition. However, visible differences were found between genotypes at 35/33 °C after continuous exposure for 3 d. Effects of heat stress were initially reflected on the leaves of sensitive genotype after 3 d heat exposure as compared to tolerant one. Level of damage such as leaf dropping, stem lodging and tip burning appeared higher in JL-3 (sensitive) as compared to PDL-2 (tolerant) under heat stress condition (Fig. 1) (Additional File 3. Fig. S3).

### Physiological and biochemical traits in response to heat stress

#### Antioxidant enzymes activity

**Figure 2:**
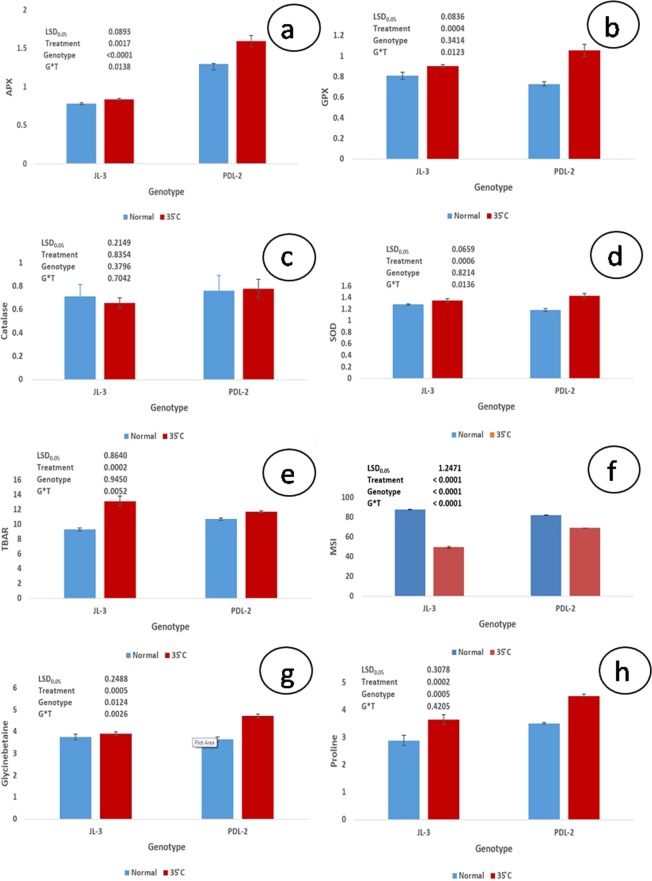
Changes in APX (µmol min^−1^ g^−1^fr.wt.) (**a**), GPX (µmol min^−1^ g^−1^fr.wt.) (**b**), CAT (µmol min^−1^ g^−1^fr.wt.) (**c**), SOD (unit min^−1^ g^−1^fr.wt.) (**d**), TBARS (µmol g^−1^fr.wt.) (lipid peroxidation) (**e**), MSI (%) (**f**) Glycine betane content (µmol g^−1^fr.wt.) (**g**), Proline content (µmol g^−1^fr.wt.) (**h**), of heat tolerant (PDL-2) and sensitive (JL-3) lentil genotypes under control and heat stress.

Activities of all the antioxidant enzymes such as APX, GPX and SOD in shoots were significantly higher in response to heat stress conditions than control in both the genotypes (Fig. 2a,b,d). However, CAT activity decreased or was found to be almost unaltered under heat stress in comparison to the control (Fig. 2c).

#### Lipid peroxidation

Lipid peroxidation level in leaves of both the genotypes, estimated in terms of malondialdehyde (MDA) content, is presented in Fig. 2e. MDA concentration in tolerant and sensitive genotypes was higher than control at 35/33 °C and particularly in sensitive (JL-3) genotype as compared tolerant (PDL-2) genotype exposed to heat stress environment.

#### Membrane stability index

MSI reduced under heat stress condition for both tolerant and sensitive genotypes. However, tolerant genotype showed higher MSI than sensitive one under heat stress condition (Fig. 2f).

#### Glycine betaine content

Significant effect of heat stress was observed on glycine betaine content in both the genotypes. In tolerant genotype PDL-2, accumulation of glycine betaine was significantly higher as compared to JL-3 under heat stress (Fig. 2g).

#### Proline content

Proline accumulated in both the genotypes in response to heat stress (Fig. 2h), where significantly higher content was accumulated in PDL-2 (p < 0.05) than JL-3. Proline concentration increased rapidly as temperature rose from 27/16 °C to 35/33 °C in tolerant genotype.

#### *In vitro* and *in vivo* pollen germination

Pollen germination reduced by 12.1% in PDL-2 (tolerant) at 34.5/23.4 °C. However, JL-3 (sensitive) showed higher reduction (33.2%) at the same level of heat stress (Additional File 4. Fig. S4 and Additional File 5. Fig. S5). *In vivo*, pollen germination was recorded in heat sensitive (JL-3) as well as heat tolerant (PDL-2) genotypes after 30 min of hand pollination under heat stress. Stressed pollens collected from PDL-2, germinated on stressed stigma of JL-3 (Additional File 6. Fig. S6) but collected stressed pollens from JL-3 did not germinate on stressed stigma of PDL-2 (Additional File 7. Fig. S7).

## Transcriptome Assembly

Illumina HiSeq 2000 Raw Fastq files were used for *de novo* assembly using Trinity de novo assembler software. Minimum number of contigs obtained was 91,926 in Tolerant Treated Rep2 while maximum number of contigs viz. 1,04,424 were obtained in Tolerant Control Rep-3. Smallest contig length filter was applied and only contigs having minimum size of 500 bp were taken. Statistics of assembly and coverage of individual samples as well as details of all the transcripts within different combinations are presented in Table 1, Additional File 19. Table S4 and Additional File 22. Excel sheet. respectively.

**Table 1:** Total raw data generated through transcriptome assembly.

Properties	Total Coverage (Gb)	Number of reads generated	Total contigs	Smallest contig length	Longest contig length	Average contig length	N_80_	N_50_	N_20_
Tolerant- Treated_1	6.1	22536460	93705	500	16470	1437	985	1770	3005
Tolerant- Treated _2	5.9	24355081	91926	500	16818	1426	978	1766	3006
Tolerant- Treated_3	5.7	23604075	90267	500	15500	1423	980	1760	2978
Sensitive-Treated_1	6.3	26210135	101161	500	16626	1465	991	1796	3092
Sensitive-Treated_2	6.1	22541528	99477	500	16182	1463	993	1797	3088
Sensitive-Treated_3	5.8	23960480	97231	500	16550	1444	983	1781	3047
Tolerant-Control_1	6.4	26685338	101480	500	15597	1456	1024	1844	3060
Tolerant-Control_2	6.0	24935410	98301	500	15365	1423	1015	1822	3038
Tolerant-Control_3	6.3	28521319	104424	500	15858	1458	1026	1832	3059
Sensitive-Control_1	7.3	30210135	95978	500	15764	1523	990	1793	3033
Sensitive-Control_2	6.8	27960145	93061	500	16470	1516	980	1755	2965
Sensitive-Control_3	7.8	32460168	94874	500	15764	1524	997	1795	3056

**Figure 3:**
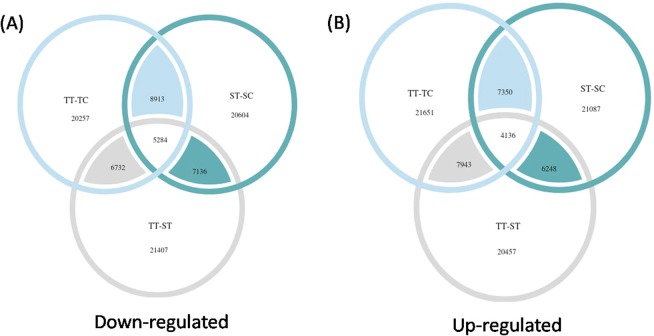
Venn diagram showing overall expression of (**A**) down-regulated (**B**) up-regulated DEGs in combination tolerant treated vs. tolerant control (TT-TC), sensitive treated vs. sensitive control (ST-SC) and tolerant treated vs. sensitive treated (TT-ST).

Differential gene expression analysis was performed between three sets of contrasting datasets, viz - tolerant treated vs tolerant control, tolerant treated vs sensitive treated, sensitive treated vs sensitive control using EdgeR software with default parameters. Wherein, TMM normalization was carried out for each set of comparison, total number of up regulated and down regulated genes in all the three comparison groups were quite similar. However, the number of significant DEGs (FDR value < 0.05) were lesser in tolerant treated vs tolerant control than in rest of the groups. Significantly expressed genes and their relative expression levels were compared across all the 3 comparison groups to find out the expression pattern of genes upon heat stress exposure in different genotypes. DEGs for different combinations of tolerant and sensitive genotypes in response to heat stress environment is summarised in Fig. 3. Output of the DEGs were analysed between the samples to assess the correlation using PCA (Additional File 8. Fig. S8).

### Identifications of SSR, SNP and Indel markers

MISA and GATK software were used for identification of SSR and SNP markers, respectively. On an average within 3 replicates of each samples, sensitive control had highest number i.e.14,986 SSRs followed by sensitive-treated (10,960), tolerant-control (10,836) and tolerant-treated (10,233) (Additional File 20. Table 5). P1 (Mono-Nucleotide) type SSRs were abundant in all the samples followed by P2 (Di-Nucleotide), P3 (Tri-Nucleotide), P4 (Tetra-Nucleotide), P5 (Penta-Nucleotide) and P6 (Hexa-Nucleotide). Penta- and hexa-nucleotide types showed similar range of SSRs. Further, average number of SSRs in different samples for tri-nucleotide type ranged from 3,132 to 3,992.

Average SNPs found within the replicates of samples were ranked from tolerant control having highest number of SNPs (17,224) followed by sensitive treated (17,167), tolerant treated (17,110) and sensitive control (13,225) along with number of Indels which ranged from 552 to 683 in all the samples (Additional File 20. Table 5).

### Functional annotation of DEGs for plot hierarchical heat map

**Figure 4:**
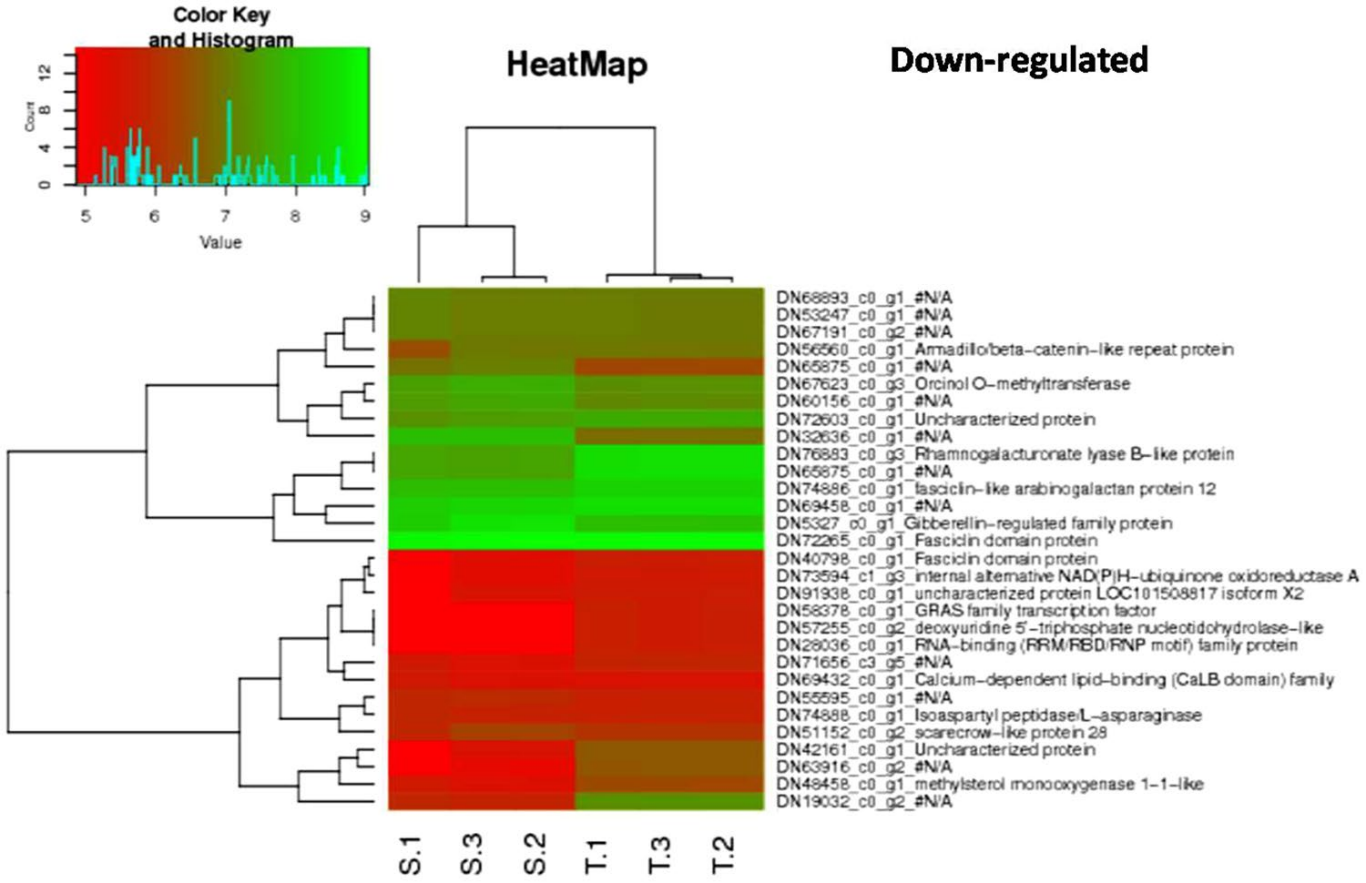
HeatMap depicting 30 down-regulated DEGs with p value < 0.05 in combination tolerant treated vs sensitive treated.

**Figure 5:**
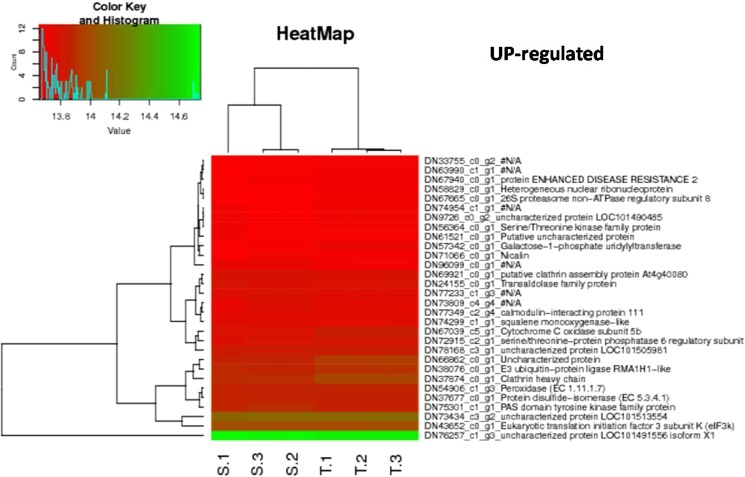
HeatMap depicting 30 up-regulated DEGs with p value < 0.05 in combination tolerant treated vs sensitive treated.

Top 30 up regulated and down regulated heat related DEGs were selected for all the major combinations as represented in Figs 4 and 5, Additional File 9. Fig. S9 and Additional File 10. Fig. S10.

In combination tolerant treated vs sensitive treated, highest log FC for up and down regulated DEGs were 9.55 and −10.59, respectively. DN68776_c2_g3, DN19596_c0_g1 and DN81013_c1_g8 were top 3 down regulated DEGs for which, first two did not match to any of the known databases and thus can be called as novel transcripts whereas, third one was annotated as Malate dehydrogenase mitochondrial gene. For upregulated DEGs, DN57816_c0_g1, DN41180_c0_g4, DN60393_c0_g4 were top 3 contigs, which were annotated as Lon protease homolog 2 peroxisomal, Probable acyl-activating enzyme 5 peroxisomal and Uncharacterized protein sll0005, respectively (Table 2). Nine hundred twenty DEGs were up regulated above log FC ≥ 3, whereas only 358 DEGs were down regulated in the combination tolerant treated- sensitive treated.

**Table 2:** Top 20 up regulated DEGs for combination tolerant treated-sensitive treated using EdgeR.

ID	LogFC	LogCPM	PValue	FDR	Description
TRINITY_DN57816_c0_g1	9.55	1.21	1E-59	4.4E-58	Lon protease homolog 2 peroxisomal
TRINITY_DN41180_c0_g4	9.37	1.12	4.11E-56	1.7E-54	Probable acyl-activating enzyme 5 peroxisomal
TRINITY_DN60393_c0_g4	9.26	0.94	8.2E-38	2.3E-36	Uncharacterized protein sll0005
TRINITY_DN10789_c0_g2	8.77	0.48	4.25E-28	9.1E-27	F-box protein At3g12350
TRINITY_DN81025_c0_g3	8.11	−0.12	1.34E-18	2.0E-17	—
TRINITY_DN29122_c0_g2	7.64	−0.45	2.16E-18	3.2E-17	Carotene epsilon-monooxygenase chloroplastic
TRINITY_DN61834_c0_g4	7.62	−0.53	7.9E-14	9.1E-13	Cinnamoyl-CoA reductase-like SNL6
TRINITY_DN70907_c0_g1	7.47	−0.63	1.78E-15	2.2E-14	Retrovirus-related Pol polyprotein from transposon TNT 1–94
TRINITY_DN62156_c0_g2	7.41	−0.71	6.36E-12	6.5E-11	Zinc finger protein JACKDAW
TRINITY_DN73377_c1_g1	7.37	2.08	1.1E-109	9.3E-108	Linoleate 13S-lipoxygenase 2-1 chloroplastic
TRINITY_DN71700_c1_g1	7.34	4.29	0	0.0E + 00	#N/A
TRINITY_DN47566_c0_g1	7.10	−0.92	1.82E-12	1.9E-11	—
TRINITY_DN36767_c0_g1	7.06	−0.99	1.98E-09	1.7E-08	—
TRINITY_DN59987_c0_g4	6.82	−1.10	6.36E-12	6.5E-11	UDP-glycosyltransferase 73C6
TRINITY_DN73578_c1_g1	6.69	3.82	0	0.0E + 00	Delta-1-pyrroline-5-carboxylate synthase
TRINITY_DN33495_c0_g1	6.67	−1.18	3.38E-12	3.5E-11	—
TRINITY_DN58346_c0_g2	6.60	−1.31	7.45E-09	6.0E-08	—
TRINITY_DN32466_c0_g2	6.60	3.62	6E-296	2.0E-293	Umecyanin
TRINITY_DN55283_c0_g2	6.58	2.22	6.4E-120	6.0E-118	Outer membrane protein Omp38
TRINITY_DN59842_c0_g2	6.55	−1.37	4.77E-07	3.2E-06	Phenylethylamine oxidase

**Figure 6:**
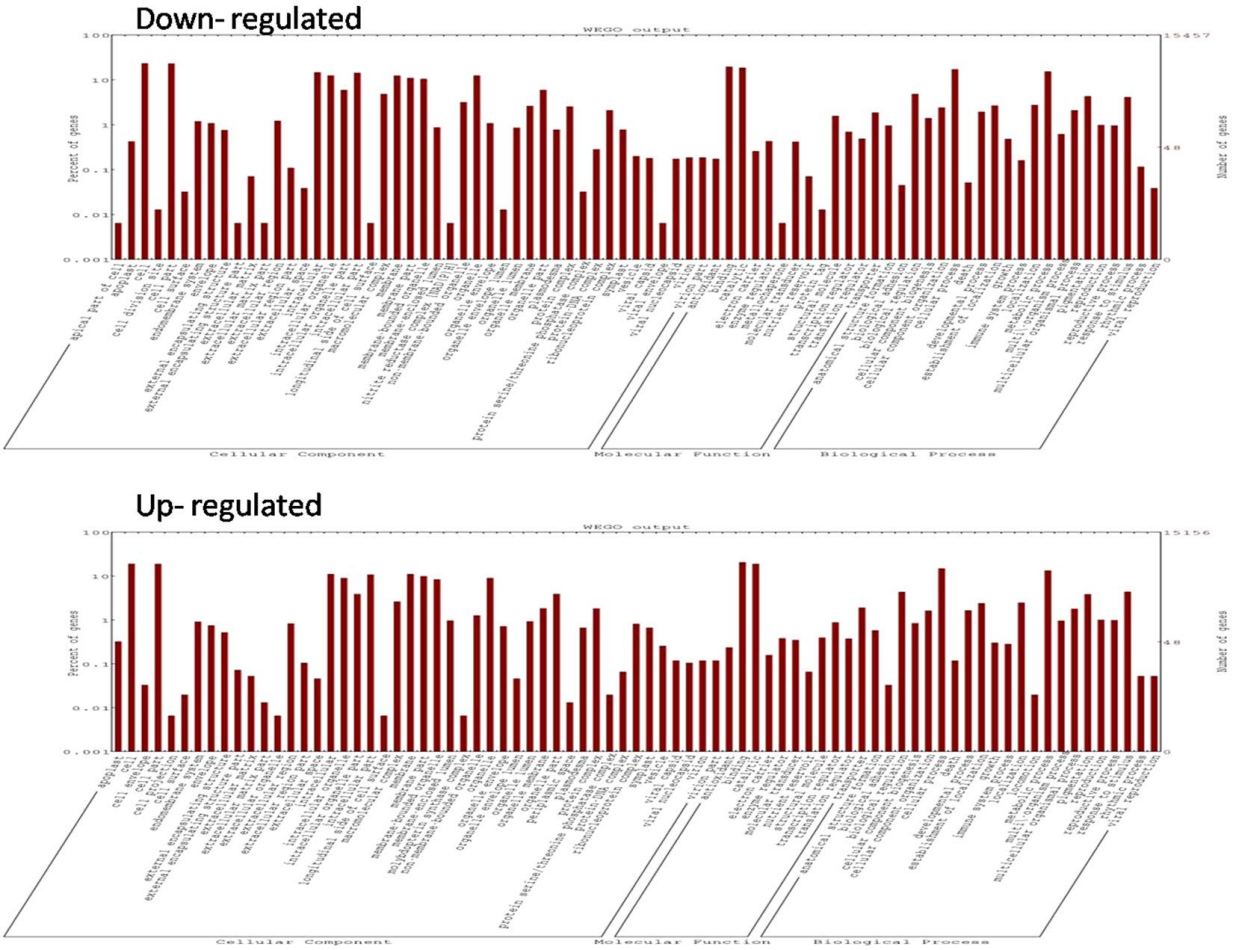
Wego plot for upregulated and down regulated GO classification in accordance to GO groups: molecular function, biological process and cellular component in combination tolerant treated vs sensitive treated under heat stress.

Upregulated and downregulated GO annotations were distributed into three categories viz. biological process, cellular component and molecular functions which were represented by WEGO plots. Upregulated and downregulated GO annotations for the combination tolerant treated vs sensitive treated revealed 15,156 and 15,457 genes, respectively (Fig. 6). Similarly, in tolerant treated vs tolerant control and sensitive treated vs sensitive control comparison groups, a total of 13,508;16,807 and 16,262;15,335 up regulated and down regulated genes were identified, respectively (Additional File 11. Fig. S11 and Additional File 12. Fig. S12). Cellular category had highest components in addition to number of genes per components followed by biological process and molecular functions.

Among all the GO terms for cellular component, maximum count of genes were associated in apoplast (GO:0048046) and cell division site (GO:0032153) in tolerant treated vs sensitive treated comparison group. These were found to be down regulated in this comparison group, whereas GO terms viz. cell (GO:0005623) and cell parts (GO:0044464) had highest number of genes which were upregulated. For molecular function GO terms, binding (GO:0005488) and catalytic (GO:0003824) which were up regulated as well as down regulated in different combinations, showed highest number of genes and for biological process, cellular process (GO:0009987) and metabolic process (GO:0008152) had highest set of genes for different combinations (Fig. 6).

**Figure 7:**
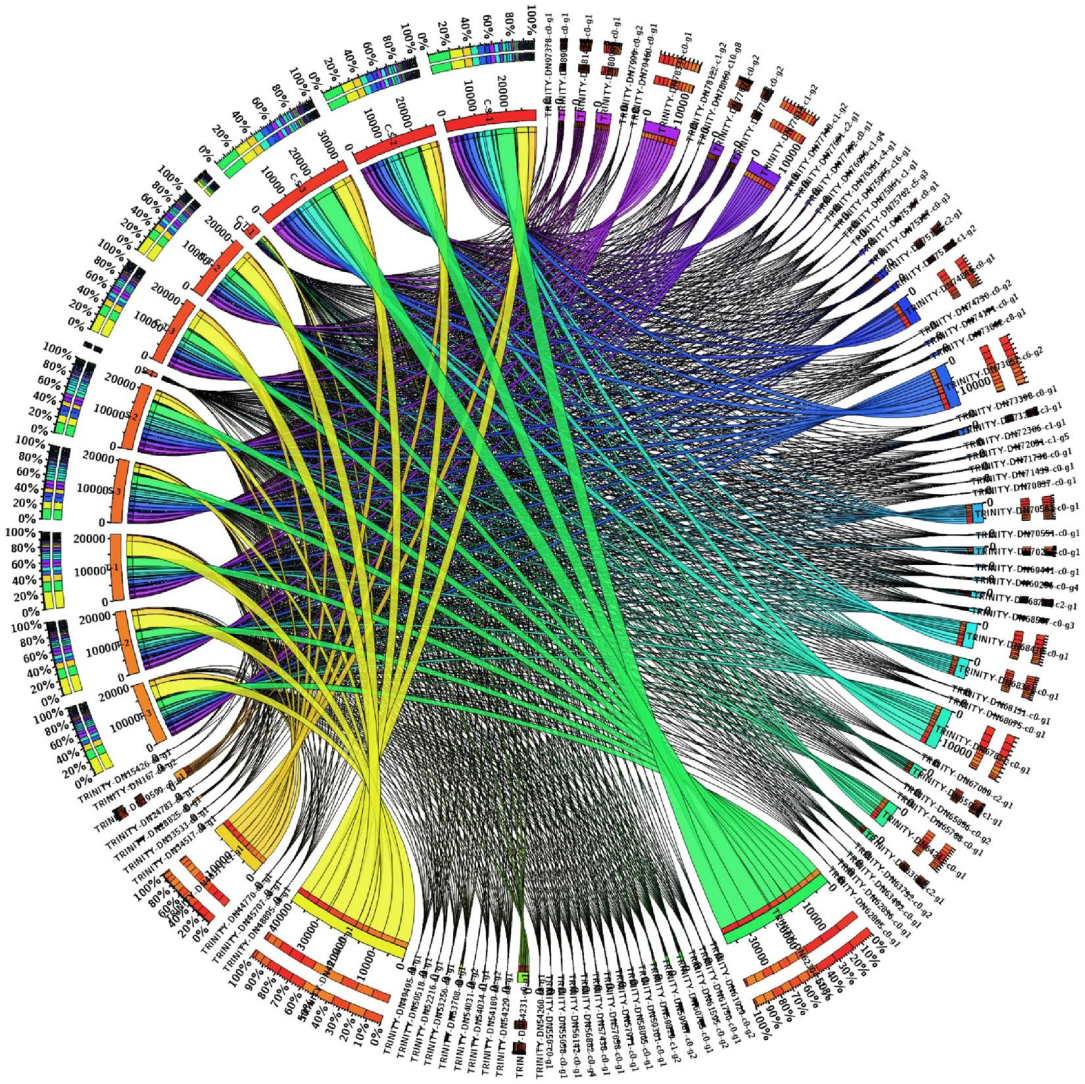
Circular plot depicting DEGs involved in tolerant vs sensitive genotypes under heat stress was plotted using Circos version 0.62. Tabular data representing genotypes in columns & DEGs values in rows has been plotted in this circular plot. Rows are represented by circularly arranged segments (outermost ring), whose length is proportional to the total cell values in a row and columns are represented by circularly arranged segments (inner ring) whose length is proportional to the total cell values in a column. Ribbons represent row & column IDs. The 3 outer rings are stacked bar plots that represent relative contribution of a cell to row and column totals. Expression value is expressed in colours with red colour representing lowest value & purple colour representing highest values.

**Figure 8:**
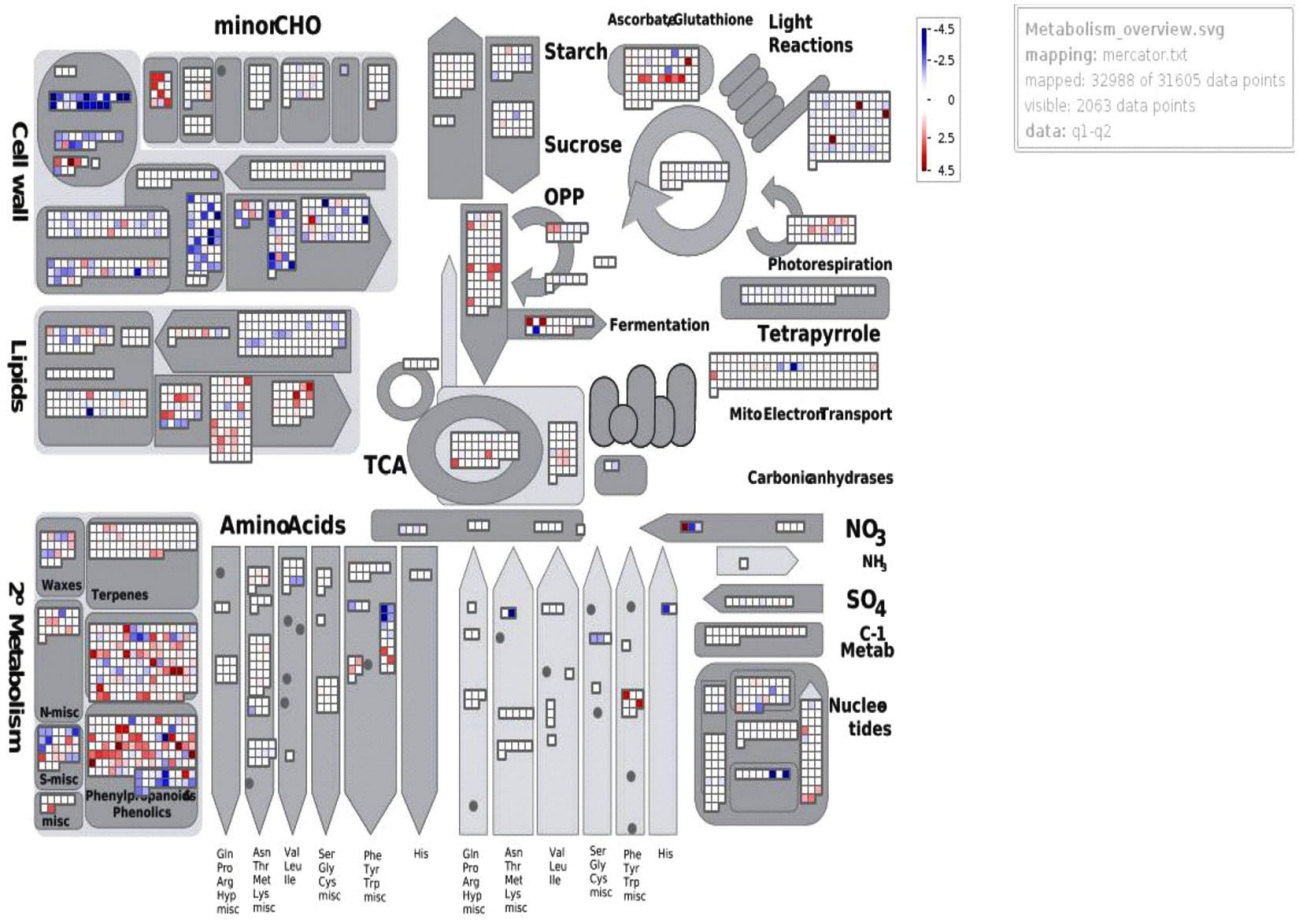
MapMan display for tolerant vs sensitive under heat stress. Up regulated genes are expressed in increased intense red while down regulated as blue at the amplitude of 4.5 to −4.5 (log2-value).

Circos plot was generated showing association of upregulated and downregulated DEGs with tolerant and sensitive genotypes under control and stressed conditions (Fig. 7). MapMan (MapManVersion 3.6.0RC1) represented overall views of metabolic pathways involved under heat stress for different combination (Fig. 8, Additional File 13. Fig. S13 and Additional File 14. Fig. S14). For the combination tolerant treated vs sensitive treated, MapMan showed involvement of nearly all the pathways. Among the major pathways, cell wall and secondary metabolite had highest number of BINs which represented the functional categories to which genes were assigned (Fig. 8).

### Validation of g-SSRs

All 55 g-SSRs gave amplification in 96 genotypes. Among which, 18 markers were found to be polymorphic. Primer 10 had highest number of allele, genetic diversity as well as Polymorphism Information Content (PIC) (Additional File 15. Fig. S15). Major allelic frequency, PIC, number of alleles, genetic diversity and heterozygosity were deduced using power marker (Additional File 21. Table S6).

### Validation of DEGs through RT-qPCR

**Figure 9:**
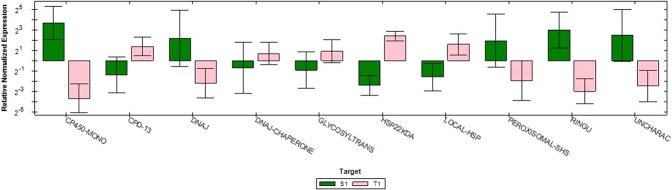
Genes relative expression profile using real time PCR. 2^-(ΔΔCT)^ method was used to obtain relative quantification using β-tubulin as reference gene. Data represents average from three biological replicates and error bars indicates standard deviation (±SD).

**Figure 10:**
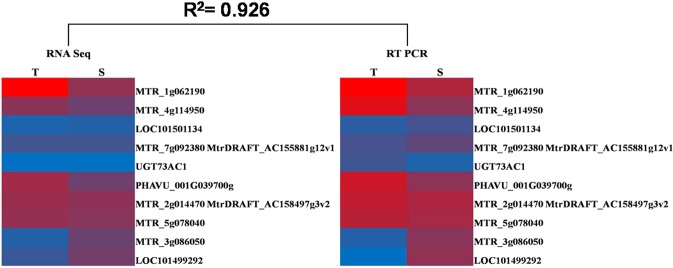
Correlation between RNA Seq and real time PCR. Log fold change is depicted through colour intensity as described in heat maps.

Ten DEGs with log FC ≥1.27 selected from the combination group of tolerant treated vs sensitive treated were validated on two samples (heat treated tolerant and sensitive) with three technical replicates using RT-qPCR. Degree of expression of genes amplified using RT-qPCR is represented in Fig. 9. Log2 fold change was deduced from the raw data and when compared to that of RNA Seq data, it exhibited a close association and concordance with differential expression results of genes under heat stress conditions (Fig. 10).

## Discussion

### Heat stress response at seedling stage

Hydroponics is cost effective, easy and empirical method for evaluating genotypes utilizing minimum space and time^1^. Use of hydroponics diminishes role of water stress during heat stress and confounding plant responses as in soil rhizosphere is also mitigated, therefore abiotic stress is accredited just because of heat stress alone^27^. Although, hydroponics does not replicate field environment where plants observe different kinds of interaction, but since here our target is to reveal molecular mechanism just because of heat stress alone, eliminating other stress factors is essential which can’t be possible under field conditions. Hence, hydroponics is one of the best approaches to reveal true genes responsible for heat stress. In present study, plants were subjected to heat stress continuous at 35/33 °C for 3 d. As a result, plants of sensitive genotype showed marked wilting of leaves along with lodging of stems when compared to plants of tolerant one (Fig. 1). This indicated that 3 d continuous heat exposure is sufficient to discriminate genotypes as tolerant or sensitive, in addition to the fact that the genotypes has already been selected from a whole spectrum of 119 genotypes by Singh *et al*.^1^. Same duration of heat stress did not affect the basic metabolism in tolerant genotype which was affected in case of sensitive genotype. This indicates that visual evaluation of plant wilting and necrosis provides a reliable method for ranking of genotypes in response to long term and/or more severe heat stress conditions. Momonoki *et al*. elucidated that wilting after thermal stress in plants like cowpea, cucumber and radish is associated with acetylcholine movement and similar to this study, they have used low degree of wilting as a parameter for distinguishing cultivars under heat stress^28^.

### Heat stress response at reproductive stage

Heat stress reduced pollen germination in tolerant and sensitive genotypes and the level of reduction was less in tolerant (PDL-2) genotype at 35/20 °C under controlled conditions. Heat condition damages pollen germination due to decrease in moisture content i.e. desiccation and soluble sugar concentration in pollen^29,30^. Reduction in pollen germination under heat stress has been reported in many crops such as rice and soybean^31,32^. Low pollen germination under heat stress could be result of carbohydrate metabolism of anthers and subsequently of their tapetum cells^33^. These results support the fact that high temperature stress affects pollen germination and consequently results in reduction in seed yield as also reported in several species earlier.

In controlled environment, stressed pollens from tolerant (PDL-2) genotype germinated on stressed stigma of sensitive genotype (JL-3). However, stressed pollens from sensitive (JL-3) genotype did not germinate on stressed stigmas of tolerant (PDL-2) genotype, indicating that pollen was fertile at 35/20 °C (Additional File 6. Fig. 6 and Additional File 7. Fig. S7). This suggests that stigma in lentil is less sensitive to heat than pollen as reported in rice by Yoshida *et al*.^34^. Several studies in crops like beans and flax seed have reported that pollen is more sensitive to high temperature than carpel^35,36^. However, impairment of pollen and stigma tissues depends upon critical temperature of the crop and degree of heat tolerance among genotypes. Every genotype possesses optimum temperature tolerance levels and if level of temperature exceeds, percentage pollen germination and maximum pollen tube length reduces. This optima has been described through bilinear regression model by Kakani *et al*. and Kakani *et al*. in groundnut and cotton genotypes, respectively^37,38^. Overall, in present study also, pollens of lentil were found to be more sensitive to heat stress as compared to stigma when observed under controlled conditions.

### Physiological and biochemical responses under heat stress

MSI was affected in both tolerant as well as sensitive genotypes under heat stress. However PDL-2 (tolerant) maintained significantly higher MSI under both control and heat stress conditions (Fig. 2f). This shows that higher MSI elevates the tolerance of the tolerant genotype by enhancing the physio-biochemical processes under heat stress environment. Quantitative trait loci (QTLs) linked with MSI under heat stress have also been mapped in wheat^39^. Higher MSI was reported to be associated with osmolytes and antioxidant activities in wheat^40^. Heat can remarkably modify the physical properties of biological membranes^41,42^. Production of osmoprotactants such as proline and glycine betaine in plants, normalizes the membranes disruption and also retains the protein conformation intact under various abiotic stresses^43,44^ like drought^7,45^, salinity^46,47^, heat^48^ etc. Present results also showed that heat tolerant genotype accumulated more proline and glycine betaine contents as compared to sensitive ones (Fig. 2g). Higher proline concentration has been reported to reduce photo damage to chloroplast thylakoid membranes through scavenging and/or reducing production of ROS^49^. Glycine betaine, another compatible solute increases in chloroplast under environmental stresses^50^. Elevated accumulation of glycine betaine content was observed in PDL-2 compared to JL-3. Similar increase in level of glycine betaine under heat stress at seedling stage has been reported in barley^51^. Hayashi *et al*. have observed that transformed *Arabidopsis* accumulated glycine betaine and exhibited enhanced tolerance to high temperature during seed germination and growth of seedlings^52^. They proposed that glycine betaine mitigates heat stress by reducing induction of heat shock proteins (Hsp) in those transgenic plants. Rasheed *et al*., testified involvement of both proline and glycine betaine in heat stress tolerance by giving external pre-treatments in sprouting sugarcane buds. Soaking of buds in proline and glycine betaine solutions markedly lowered hydrogen peroxide production, boosted building up of soluble sugars and shielded developing tissues for heat stress effects^53^.

MDA has been considered as physiological marker and its concentration varies under abiotic stress conditions^54,55^. In present study its concentration was found to be higher in both tolerant and sensitive genotypes when compared to their respective control samples, indicating lipid injury in tissues of both the genotypes under heat stress. Lipid peroxidation indicates the prevalence of free radical reactions occurring in tissues which reflects a correlation between heat and oxidative stress^56^. Degree of lipid peroxidation was lower in shoots of tolerant (PDL-2) genotype (Fig. 2e) as compared to sensitive one. Similar results were found in fababean, wheat and creeping bent grass under heat stress^57–59^. Induction of activities of antioxidant enzymes and metabolites can play a crucial role in plant’s resistance to heat and other abiotic stresses^60,61^. Antioxidant enzymes are considered as the first line of defence used by plants to scavenge superoxide radicals into H_2_O_2_ and O_2_. It is reported that production of H_2_O_2_ also contributes in transduction of heat signal into expression of HSPs^62^. Plants also use peroxidases like APX, CAT etc. and antioxidant enzymes to scavenge ROS. Heat stress showed a marked effect on SOD, APX, GPX and CAT activities in both tolerant and sensitive genotypes. Tolerant genotype exhibited a sharp rise in SOD, APX and GPX activities compared to sensitive one (Fig. 2). Less increase in lipid peroxidation level in tolerant genotype could be a result of higher activities of SOD, APX and GPX enzymes (Fig. 2). PDL-2 exhibited no significant effects on CAT activity under heat stress, while its activity decreased significantly in JL-3 suggesting that tolerant genotype had experienced lower level of oxidative stress due to induced antioxidant enzymes activities and detoxified ROS. Similar to our study, increase in SOD activity of temperature tolerant genotypes was reported in wheat, although contrary to our results, activity of CAT was also found to be increased in their study^63^. Elevated SOD, APX and GPX activities were observed in lily plants under heat stress together with reduction in CAT activity^64^. Reduction in CAT activity was also reported in tomato plants which were subjected to 35 °C heat stress^65^.

### Transcriptome analysis and stress responsive genes under heat stress

Next Generation Sequencing (NGS) was useful in identification of novel genes correlated with biotic and abiotic stresses^66,67^. *De novo* assembly has been used on RNA Seq data for mining out novel genes in several crops like wheat, *Vicia faba* etc.^68,69^. Understanding of underlying molecular mechanism of heat tolerance is still unknown in lentil. However, some reports shedding light on molecular mechanisms of other abiotic stresses like drought and cold are available^7,70^. Transcriptome analysis may provide an overview of novel genes and regulatory networks linked with heat tolerance mechanism in lentil. Previous studies on transcriptome analysis in lentil have provided valuable information and genomic insight for different aspects such as identification of expressed sequenced tags (ESTs) derived SSR and SNP markers^7,71,72^. Also, heat stress responsive genes have been identified for several crops like *Arabidopsis*, wheat etc., using transcriptomic analyses^73,74^.

In the present study, a transcriptome comparison of heat sensitive (JL-3) and heat tolerant (PDL-2) genotypes was attempted to establish potential candidate genes involved in response to heat stress in lentil. cDNA samples of both genotypes were sequenced and in total, 23,605,081 to 32,460,145 clean reads were used for *de novo* assembly, generating 90,267 to 104,424 contigs for different combinations (Table 1). Number of contigs generated in this study is comparable or similar to other studies^7,71,72^. Tolerant genotype (PDL-2) showed increased number of DEGs as compared to sensitive genotype at 3 d of continuous heat stress, including both up and downregulated genes. Total range of 13,510 to 16,720 upregulated and 15,141 and 16,817 downregulated genes were found in different combinations (Fig. 3). Results for total DEGs deduced under heat stress, were much higher as compared to that of wheat (6,560), switchgrass (5,365) and Chinese cabbage (625)^74–76^.

It was found that most of DEGs were mainly confined to the cell wall and secondary metabolic components (Fig. 8). BINs which showed significant up and down regulation in the combination tolerant treated vs sensitive treated were also visualized under cell wall and secondary metabolism. Changes in BINs related to cell wall have been reported in barley caryopses under heat stress by Mangelsen *et al*.^77^. Modification of cell wall under abiotic stress would prevent plant from severe damage^78,79^. Genes encoding diverse cell wall enzymes like arabino galactan protein, cellulose synthase, expansin etc. were significantly up regulated following high temperature stress in *Brassica rapa *L^75^. In the present investigation, contig DN88332_c0_g1 (Plasmodesmata Callose-Binding Protein 3 (PDCB) up regulated (log FC = −7.67) in tolerant treated vs sensitive treated was localized in the plasmodesmata neck region that gives structural anchor between plasma membrane component of plasmodesmata and cell wall. Increased *PDCB1* elevates callose accumulation that enhances correlation between PDCB-mediated callose deposition and plant’s cell-to-cell communication^80,81^. Callose accumulation has been found to be beneficial for plants in many important aspects such as plasmodesmata regulation and avoiding biotic and abiotic stresses through multifaceted defence response controlled by distinct signalling pathways^82^. Detectable callose deposition appears within minutes of impairment caused by various stresses including raised temperatures. Callose plugs at plasmodesmata are involved in maintenance of dormancy by isolating meristem from symplastic continuity with neighbouring tissues^82^. Rinne *et al*. has proposed a dormancy cycling model to depict subsequent states of cellular transmission in meristem with attributed sensitivities to different abiotic stresses^83^. Further, it was reported that callose synthase was needed for exine formation during micro gametogenesis and pollen viability in *Arabidopsis*^84^. Also, callose is crucial for pollen wall patterning but not pollen tube growth^85^. Callose deposition (GO: 0052542) and callose localization (GO: 0052545) were found to be involved in heat stress response of sweet maize varieties through transcriptome analysis^86^. DN70092_c0_g4 (log FC = 1.94) Phosphatidylinositol/phosphatidylcholine transfer protein SFH13 (Sec Fourteen Homolog 13); DN59454_c0_g1(log FC = 2.02) CDP-diacylglycerol–glycerol-3-phosphate 3-phosphatidyltransferase 1 chloroplastic; DN72602_c0_g1(log FC = 1.52) Probable glycerol-3-phosphate acyltransferase 2 (GPAT2); DN73858_c0_g1(1.34) O-acyltransferase WSD; DN92437_c0_g1(−1.21786624590465) Phosphatidylcholine diacylglycerol choline phosphotransferase 1 were noticed to be upregulated in tolerant genotype under heat stress. These were found to be directly or indirectly involved in formation of glycerolipids. These lipid species were found to get induced in response to heat stress conditions in *Arabidopsis*^87^. Phosphatidylinositol/phosphatidylcholine transfer protein SFH13 is needed for transport of secretory proteins from golgi complex and that of phosphatidylinositol/phosphatidylcholine between membranes *in vitro*^88^. CDP-diacylglycerol–glycerol-3-phosphate 3-phosphatidyltransferase 1 is an integral membrane protein which is involved in glycerophospholipid metabolism. This protein hold high activity with CDP-dipalmitoylglycerol and low activity with CDP-dioleoylglycerol^89,90^. GPAT2 mediates initial step of glycerolipid biosynthesis which involves esterification of acyl-group from acyl-ACP to sn-1 position of glycerol-3-phosphate^91^. Various isoforms of GPAT have been classified in yeast animal and plant cells^92,93^. Zheng *et al*. has reported pivotal role of *Arabidopsis ATGPAT 1* in pollen development^91^. OsGPAT3 has been reported to be involved in anther development and male fertility in rice^94^. In present study, since GPAT2 is upregulated in tolerant genotype, at later stages of plant development, it might be involved in anther development in lentil as well. This can be further confirmed through mutation studies in lentil. GPAT enzyme has been partially purified from chilling tolerant plants of spinach and pea^95^. O-acyltransferase WSD catalyzes the terminal step in triacylglycerol synthesis by using diacylglycerol and fatty acyl CoA as substrates^96^. Diacylglycerol acetyltransferase WSD1 is required for stem wax ester biosynthesis in *Arabidopsis*^97^ whereas, Phosphatidylcholine diacylglycerol cholinephosphotransferase 1 is involved in triacylglycerol synthesis pathway^98,99^. Triglycerols are widely found as carbon and energy reserve in pollen grains. It is known that these surface lipids are required for male fertility and productive pollen-pistil interactions. A layer of lipophilic material called tryphine is embedded in exine of pollen grains. This tryphine includes small lipid bodies that primarily contains wax monomers and is responsible for reducing water loss from pollen grains. The normal exine coat plays signalling role in pollen-pistil interactions^100^. Therefore, O-acyltransferase WSD might be upregulated in tolerant genotype to protect this normal exine coat in later reproductive stage of life cycle in response to heat stress. Secondary metabolites play important roles in plant survival under heat stress^101^. Several pathways like phenyl propanoids, shikimate, mevalonate or erthritol phosphate pathway (MEP) are involved in secondary metabolite production^102^. Contig DN32591_c0_g2 (Pyruvate phosphate dikinase chloroplastic) down regulated in tolerant-sensitive treated with log FC = −8.04 catalyses the phosphorylation of pyruvate in presence of ATP to produce phospho enolpyruvate (PEP). Both cytosolic and plastidiciso-forms found in higher plants are induced under drought and salt stress^103^. In case of *Nicotiana tabacum* L. under drought condition, PPDK increased upto 2.7 folds^104^. PEP is an essential compound for Shikimate pathway in which it is converted into chorismate, a central branch point metabolite that is converted to secondary metabolites^105^. Microarray analysis in *Arabidopsis* has shown that there is a correlation between *PPDK* and *AtRP2* in pollen which has led to an opinion that *AtRP2* plays crucial part in pollen development by regulatory inactivation of PPDK^106^. This suggests that here in lentil under heat stress, down regulation of PPDK might have some correlation to pollen development later at reproductive stages.

In the present study, to identify similarity and differences in molecular mechanisms, transcriptome profiles were compared to publicly available proteome datasets of legume species for similarity percentage. *Medicago sativa* (~95.8%) showed highest similarity percentage followed by *Cajanus canjan* (~95.2%), *Cicer arietinum* (~94.9), *Lotus japonicas* (~93.8%) and least similarity was observed with *Glycine max* (~92%). Kaur *et al*. reported 12,639 unique hits matches with *Medicago truncatula* and 20,419 unique hits with *G*. *max*^107^. Therefore, number of genes along with its transcript coverage that represent EST collection are important objectives which require completely annotated reference genome sequence^107^.

Gene ontology analysis showed number of DEGs involved in apoplast were downregulated in tolerant treated vs sensitive treated genotypes whereas cell division, binding & catalytic GO terms were up regulated (Fig. 6). Cytochrome P450 enzymes which are associated with oxidative metabolism of various exogenous and endogenous lipophilic compounds were found to be down regulated in heat tolerant genotype. This was also reported in rice under heat stress^108^. Similarly *GmCYP82A3* which is a soybean cytochrome P450 family gene participates in jasmonic acid and ethylene signalling pathways and thereby have roles in providing tolerance to biotic and abiotic stresses^109^. Hsps play paramount role in protecting plants against stresses by reconstructing normal protein conformation and ultimately cellular homeostasis^110^. Data from genome wide expression profile of *Arabidopsis* plants exposed to heat stress has appreciably expanded our information on Hsps^111^. Extreme heat stress may cause protein denaturation *in vivo* and accumulation of misfolded proteins activates HSFs by inducing the disengagement of HSF and HSP70 and/or HSP90 complexes^112^. HSP70 and/or HSP90 are called up to repair protein damage. Transcriptional profiling of *Arabidopsis* Hsps and Hsfs has revealed extensive cross talk between heat and non-heat stress response pathways^113^. In present investigation, many Hsps were induced during heat stress in PDL-2 (tolerant) genotype, confirming role of upregulated Hsps in providing tolerance to PDL-2. Overall GO enrichment analysis showed 15,156 up and 15,457 down regulated GO terms in the combination tolerant-sensitive which were much higher when compared to that of chinese cabbage and switch grass in response to heat stress^75,114^. Biological membranes circumscribe sensory aids competent of perceiving specific signals and transducing them into apt gene expressions^115^. In present study, plasma membrane and cell wall were affected under heat stress which might have triggered interaction of signal molecules at one or more specific sites resulting in changes in anabolic and catabolic processes. These results were similar to that of heat exposed chinese cabbage and switch grass^75,114^.

Contigs DN57816_c0_g1 and DN41180_c0_g4 with log FC of 9.54, 9.36 and gene descriptions as Lon protease homolog 2 peroxisomal and Probable acyl-activating enzyme 5 peroxisomal, respectively were top two up regulated in the combination tolerant treated vs sensitive treated (Table 2). These were confined to sustained matrix protein import into peroxisomes; transporter for β-oxidation during germination and establishment; jasmonic acid biosynthesis and conversion of indole butyric acid to indole acetic acid. Lon protease homolog 2 peroxisomal is serine protease and helps in selective break down of polypeptides in the peroxisomal matrix. Deficiency of Lon 2 in *Arabidopsis* has resulted in enhanced peroxisomal degeneration by autophagy and there by peroxisomal proteins was accumulated in the cytosol^116^. Probable acyl-activating enzyme 5 peroxisomal may act as an acid-thiol ligase that stimulates carboxylic acids by forming acyl-CoAs. OPCL1 peroxisomal acyl-activating enzyme was found to be involved in jasmonic acid biosynthesis in *Arabidopsis*^117^. An acyl activating enzyme is also reported to be involved in formation of precursor for cannabinoid biosynthesis in *Cannabis sativa* trichomes^118^. Further, top two down regulated contigs DN68776_c2_g3 and DN19596_c0_g1 with log FC −10.58 and −9.45, respectively had no gene descriptions. Though, first one had protein description of Protein detoxification (Multidrug and toxic compound extrusion protein (MATE), AT3G26590.1) but later has no annotation. MATE transporters are conserved transporter families in nature which are involved in the maintenance of homostasis by excretion of waste products and xenobiotics^119^. Wang *et al*. have demonstrated that MATE gene family has expanded via tandem and segmental duplication in rice and *Arabidopsis*^120^.

Contigs annotated as peroxidase 15, 42, (DN56326, DN54906) viz. Glyoxylate/succinic semialdehyde reductase 2 chloroplastic and Cytochrome P450 (DN54719) were upregulated in tolerant genotype against sensitive genotype under heat stress conditions with Log FC above 5. Peroxidases are involved in scavenging superoxide radical, toxic reductant’s oxidation, lignin metabolism, catabolism of auxin and oxidative stress^121,122^. Glyoxylate/succinic semialdehyde reductase 2 is involved in detoxification of reactive aldehydes, glyoxylate and succinic semialdehyde into glycolate and γ hydroxyl butyrate, respectively and ultimately functions in maintenance of redox homeostasis^123,124^. Cytochrome P450 is involved in removal of ROS and synthesis of secondary metabolites such as stilbenoid, diarylhepatanoid and gingerol^109^. During physiological analysis of genotypes under heat stress also, tolerant genotype has exhibited a sharp rise in peroxidases like SOD, APX and GPX activities compared to sensitive one. Detection of increase in antioxidant activity in tolerant genotype due to heat stress in the present study reaffirms the transcriptomics evaluation. Similarly, expressions of several other physiological genes were altered under heat stress in tolerant genotype as compared to sensitive one. In this class of genes, those which were highly upregulated were Delta-1-pyrroline-5-carboxylate synthase (P5CS), 36.4 kDa proline-rich protein, Respiratory burst oxidase homolog protein C and D, Proline transporter 1 and Ornithine amino transferase mitochondrial. Delta-1-pyrroline-5-carboxylate synthase is the rate-limiting enzyme for proline biosynthesis and undergo feedback inhibition by proline which is lost in plants due to stress conditions^125^. In the present study, up-regulation of this enzyme under heat stress reflects tolerant genotype strategy to avoid feedback inhibition by proline, so that more proline can be synthesized under heat stress, which is true as per physiological analysis. Similarly, Pyrroline-5-carboxylate (P5C) is a common intermediate in the synthesis and metabolism of proline, which is produced by ornithine aminotransferase (OAT). This enzyme functions in an alternative proline metabolic pathway of mitochondria in response to stress conditions^126^. Up regulation of OAT represents operation of alternative proline metabolic pathway under heat stress in mitochondria of tolerant genotype. DEGs which were downregulated included Purple acid phosphatase 17, Cationic peroxidase 1, Proline-tRNA ligasechloroplastic/mitochondrial, Proline-rich receptor-like protein kinase PERK1 and Hydroxyproline O-arabinosyltransferase 1.

When expression of transcription factors between heat tolerant and sensitive genotypes for heat stress were compared, expression was found higher in PDL-2 compared to JL-3. ABA responsive protein (DN22374_c0_g1), WRKY transcription factor WRKY24 (DN10173_c0_g1), DnaJ homolog subfamily B member 13 (DN73300_c5_g2), 17.1 kDa class II heat shock protein (DN96073_c0_g1) were some of the well-known stress responsive genes which were common in both the genotypes with Log FC of 3.12, 2.81, 2.90, 3.90, respectively. Similar findings were reported in barley caryopses, Chinese cabbage and switch grass under heat stress conditions^75,77,114^. Signalling by stress phytohormone ABA is involved in acquired thermo tolerance^127,128^. Wang *et al*., revealed that HSFA2 is associated in ABA mediated heat stress tolerance in tall fescue and *Arabidopsis*^129^. Liu *et al*., reported that ABA can regulate the expression of several small Hsps (sHsps) in maize leaves when imposed to combined drought and heat stresses^130^. In the present study, NAC and WRKY transcription factors were found over-expressed in PDL-2 compared to JL-3. NAC has already been reported to be involved in heat stress in *Arabidopsis*, rice and wheat^131–133^. Whereas implication of WRKY25 in thermo tolerance in *Arabidopsis* was demonstrated by Li *et al*.^134^.

### Validation of g-SSRs

Out of 55 g-SSRs, 18 were found polymorphic (32.72%) while genotyping against 96 genotypes. All the markers showed amplification indicating the presence of marker locus within the lentil genome. These markers can be used for QTL identification and molecular assortment of species and can be further utilized for varied applications in lentil breeding. Similar to this study Hou *et al*. has also used transcriptomic analysis for identification of gSSR markers in proso millet. They also validated identified gSSRs by detecting polymorphism in 56 accessions^135^.

### Validation of DEGs

To confirm credibility of DEGs obtained in this study, 10 DEGs belonging to different gene families, (including 5 upregulated and 5 downregulated in tolerant-sensitive combination) were selected to examine their expression patterns via RT-qPCR (Additional File 17. Table S3). RT-qPCR results showed that expression of all these DEGs were similar to those obtained from Illumina sequencing analysis (Figs 9 and 10). Besides, the fold changes obtained by DEGs were generally higher than those obtained by RT-qPCR, which was a comprehensive phenomenon in some studies. These findings showed that the method used to ascertain DEGs in this study was valid. Up regulated DEGs validated through RT-qPCR analysis were MTR_1g062190 (Cytochrome P450 monooxygenase), MTR_4g114950 (ABC transporter domain protein), PHAVU_001G039700g (Uncharacterized protein), MTR_2g014470 [DnaJ homolog subfamily B member 4 (DNAJB4)] and MTR_5g078040 (peroxisomal small heat shock protein). Cytochrome P450-dependent monooxygenases represents a large group of heme-containing enzymes, where classical forms catalyzes NADPH- and O_2_-dependent hydroxylation reactions. Non-classical forms are monogeneses that either do not employ flavoproteins for dioxygen activation or fail to integrate molecular oxygen in their concluding product. Cytochrome P450-dependent mono oxygenases are involved in biosynthetic or detoxification pathways^136,137^. Plant ABC transporter proteins play paramount role in plant growth and development processes. These are involved in detoxification, chlorophyll biosynthesis, arrangement of Fe/S clusters, stomatal movement and ion fluxes^138^. *STAR1* and *STAR2* are bacterial type ABC transporters found to be involved in aluminium stress tolerance in rice^139^. OsABCG15, an ABC transporter gene was found to play crucial role in anther cuticle and pollen exine development in rice^140^. DNAJB4 belongs to heat shock protein (Hsp 40) family. sHsps are widespread molecular chaperons. Plants employ these Hsps in peroxisomal matrix to avoid undetailed gathering of partially denatured proteins under both physiological and stress conditions^141^. Similar to this study, expression of AtHsp15.7, which is a peroxisomal located sHsp is reported to be induced under heat and oxidative stress in *Arabidopsis*^141^. Earlier studies have shown that considerable tolerance to drought and high light can be attained by over-expressing cytosolic or plastidic heat-shock proteins^142,143^. Validated DEGs which were downregulated in RT-PCR analysis includes LOC101501134 [Cytochrome P450 superfamily protein (CPD 13)], MTR_7g092380 (DNAJ chaperone), UGT73AC1 (β glucosyltransferase), MTR_3g086050 (localized small heat shock protein) and LOC101499292 (HSP 22 KDa). Glycosyl transferases constitute indispensable multigene family present in all organisms. In plants they have role in glycosylation of plant products which can help them in survival under adverse conditions^144^. Expression of UGTs was reported to be localized in parts of expeditiously dividing cells^145^. High expressions of UGTs are involved in acute cell division which indicates involvement in cell cycle regulation^145^.

## Supplementary information

Grouped Supplymentary file

Additional file 22

## Data Availability

The dataset for two genotypes supporting the conclusions of this article is deposited in Sequence Read Archive repository (SRA) with SRA submission ID – SUB3390924. [http://www.ncbi.nlm.nih.gov/sra].
