# Supplementary material for: Genome wide transcriptome analysis reveals vital role of heat responsive genes in regulatory mechanisms of lentil (Lens culinaris Medikus)
Source: Sci Rep. 2019 Sep 10;9:12976. doi: 10.1038/s41598-019-49496-0 (PMC6736890; doi:10.1038/s41598-019-49496-0)
Supplement: Supplementary file 1 — Grouped Supplymentary file [file 41598_2019_49496_MOESM1_ESM.pdf]

Genome wide transcriptome analysis reveals vital role of heat responsive genes in regulatory mechanisms of lentil (*Lens culinaris* Medikus)

Dharmendra Singh[1*], Chandan Kumar Singh[1], Jyoti Taunk[1], Vasudha Singh[1], Madan Pal[2*] & Kishor Gaikwad[3]

[1]Division of Genetics, ICAR-Indian Agricultural Research Institute, New Delhi-110012

[2]Division of Plant Physiology, ICAR-Indian Agricultural Research Institute, New Delhi-110012

[3]ICAR-National Research Centre on Plant Biotechnology, Pusa Campus, New Delhi-110012

*Corresponding authors emails: dharmendrapbg@rediffmail.com; madanpal@yahoo.com

Additional file 1: Fig. S1. Flow chart of samples used for extraction of RNA under control and heat stressed plants.

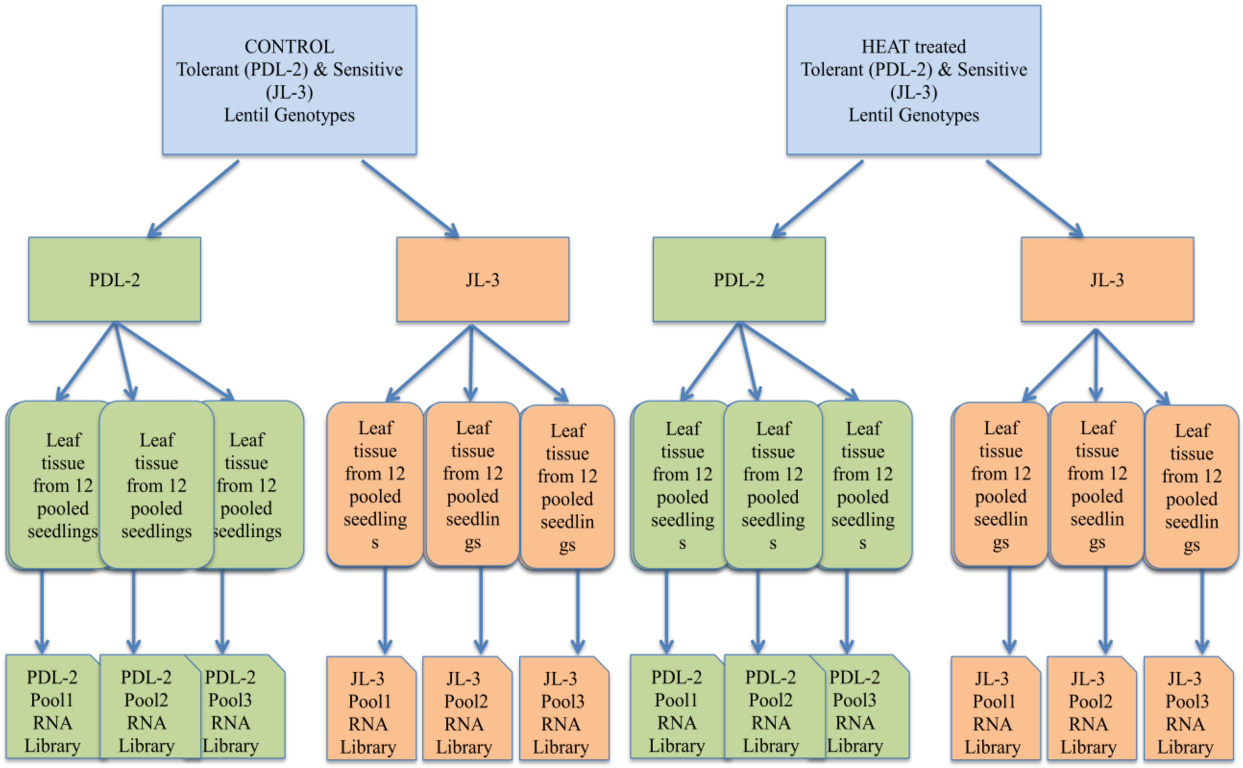

Genome wide transcriptome analysis reveals vital role of heat responsive genes in regulatory mechanisms of lentil (*Lens culinaris* Medikus)

Dharmendra Singh[1*], Chandan Kumar Singh[1], Jyoti Taunk[1], Vasudha Singh[1], Madan Pal[2*] & Kishor Gaikwad[3]

[1]Division of Genetics, ICAR-Indian Agricultural Research Institute, New Delhi-110012

[2]Division of Plant Physiology, ICAR-Indian Agricultural Research Institute, New Delhi-110012

[3]ICAR-National Research Centre on Plant Biotechnology, Pusa Campus, New Delhi-110012

*Corresponding authors emails: dharmendrapbg@rediffmail.com; madanpal@yahoo.com

Additional file 2. Fig. S2 Flow chart representing work flow used for generation as well as annotation of contigs from different samples.

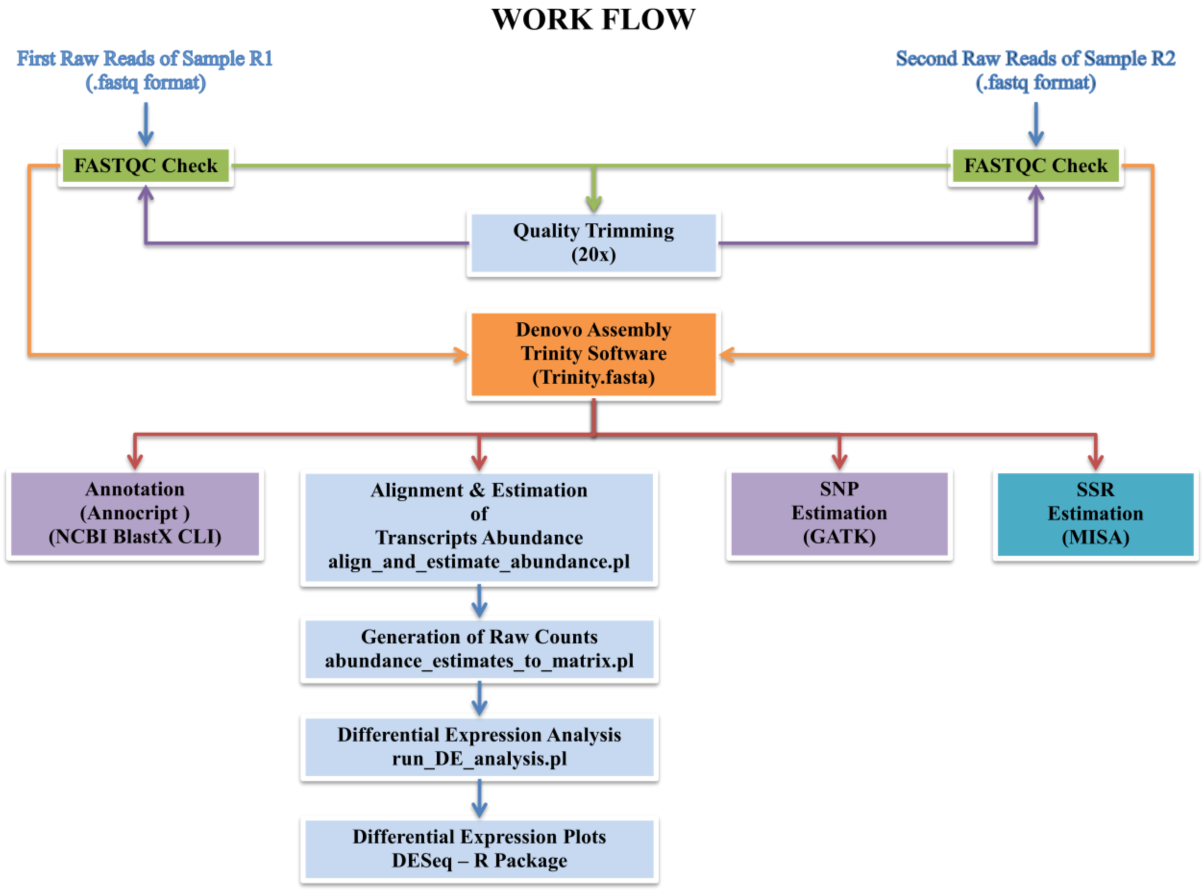

Genome wide transcriptome analysis reveals vital role of heat responsive genes in regulatory mechanisms of lentil (*Lens culinaris* Medikus)

Dharmendra Singh[1*], Chandan Kumar Singh[1], Jyoti Taunk[1], Vasudha Singh[1], Madan Pal[2*] & Kishor Gaikwad[3]

[1]Division of Genetics, ICAR-Indian Agricultural Research Institute, New Delhi-110012

[2]Division of Plant Physiology, ICAR-Indian Agricultural Research Institute, New Delhi-110012

[3]ICAR-National Research Centre on Plant Biotechnology, Pusa Campus, New Delhi-110012

*Corresponding authors emails: dharmendrapbg@rediffmail.com; madanpal@yahoo.com

Additional file 3: Fig. S3. Heat score of PDL-2 (tolerant) and JL-3 (sensitive) under heat stress conditions.

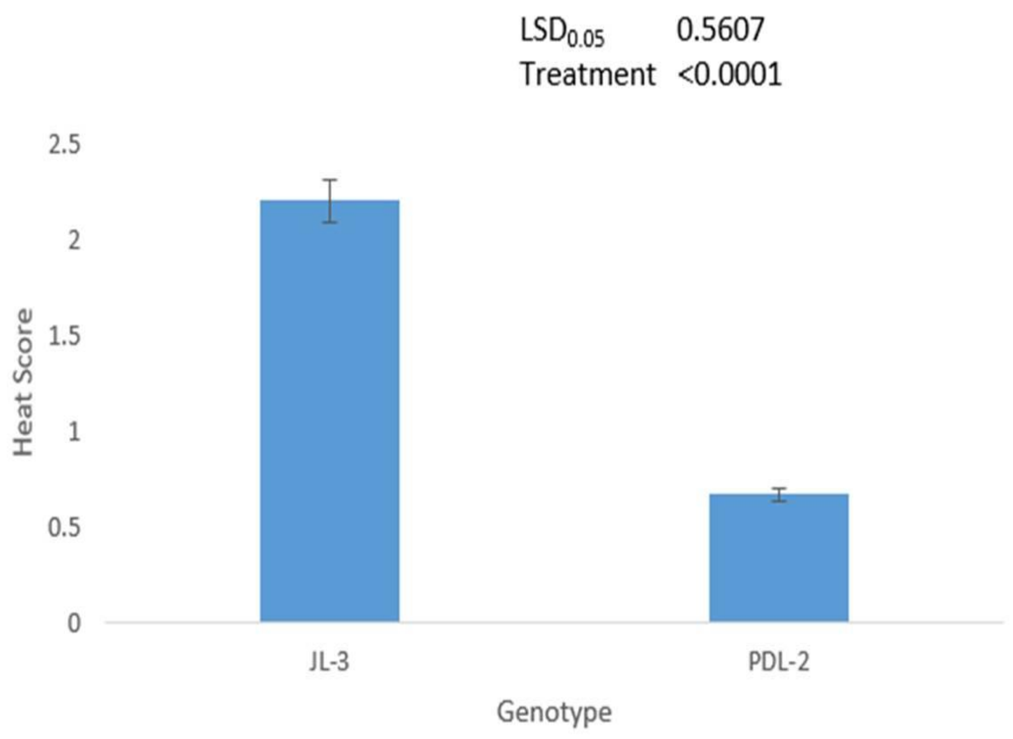

Genome wide transcriptome analysis reveals vital role of heat responsive genes in regulatory mechanisms of lentil (*Lens culinaris* Medikus)

Dharmendra Singh[1*], Chandan Kumar Singh[1], Jyoti Taunk[1], Vasudha Singh[1], Madan Pal[2*] & Kishor Gaikwad[3]

[1]Division of Genetics, ICAR-Indian Agricultural Research Institute, New Delhi-110012

[2]Division of Plant Physiology, ICAR-Indian Agricultural Research Institute, New Delhi-110012

[3]ICAR-National Research Centre on Plant Biotechnology, Pusa Campus, New Delhi-110012

*Corresponding authors emails: dharmendrapbg@rediffmail.com; madanpal@yahoo.com

Additional file 4: Fig S4. *In vitro* pollen germination in PDL-2 (tolerant) and JL-3 (sensitive) genotypes under high temperature stress (35/20^{0}C) and under controlled conditions.

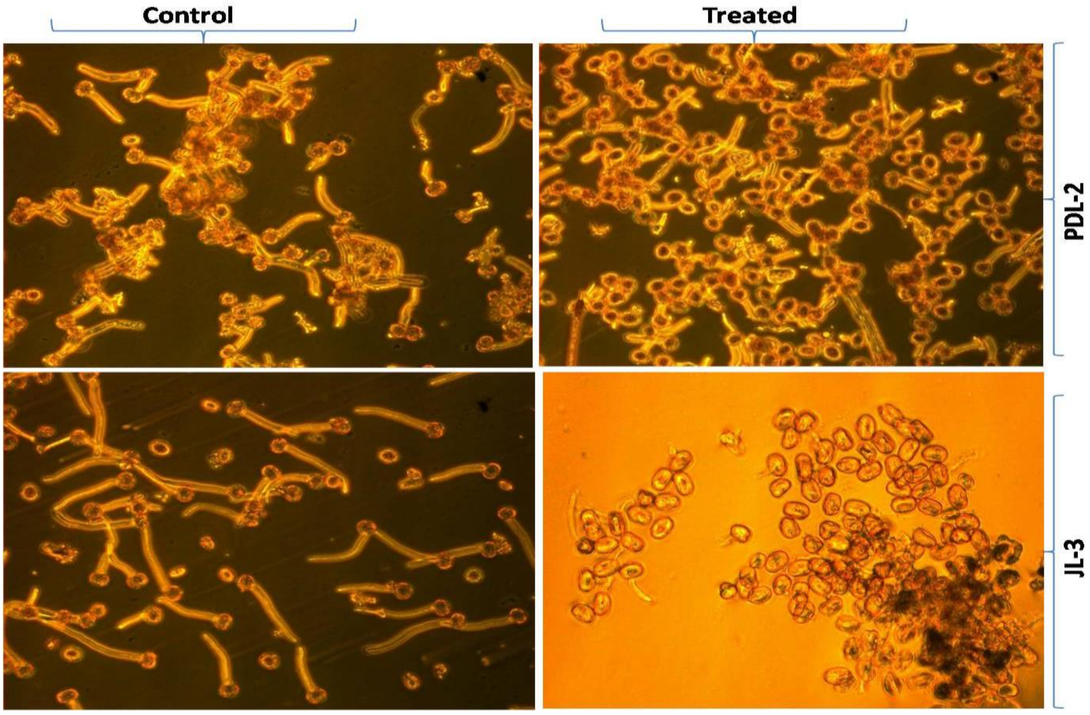

Genome wide transcriptome analysis reveals vital role of heat responsive genes in regulatory mechanisms of lentil (*Lens culinaris* Medikus)

Dharmendra Singh[1*], Chandan Kumar Singh[1], Jyoti Taunk[1], Vasudha Singh[1], Madan Pal[2*] & Kishor Gaikwad[3]

[1]Division of Genetics, ICAR-Indian Agricultural Research Institute, New Delhi-110012

[2]Division of Plant Physiology, ICAR-Indian Agricultural Research Institute, New Delhi-110012

[3]ICAR-National Research Centre on Plant Biotechnology, Pusa Campus, New Delhi-110012

*Corresponding authors emails: dharmendrapbg@rediffmail.com; madanpal@yahoo.com

Additional file 5: Fig S5. Pollen germination of PDL-2 (tolerant) and JL-3 (sensitive) under controlled and heat stress conditions.

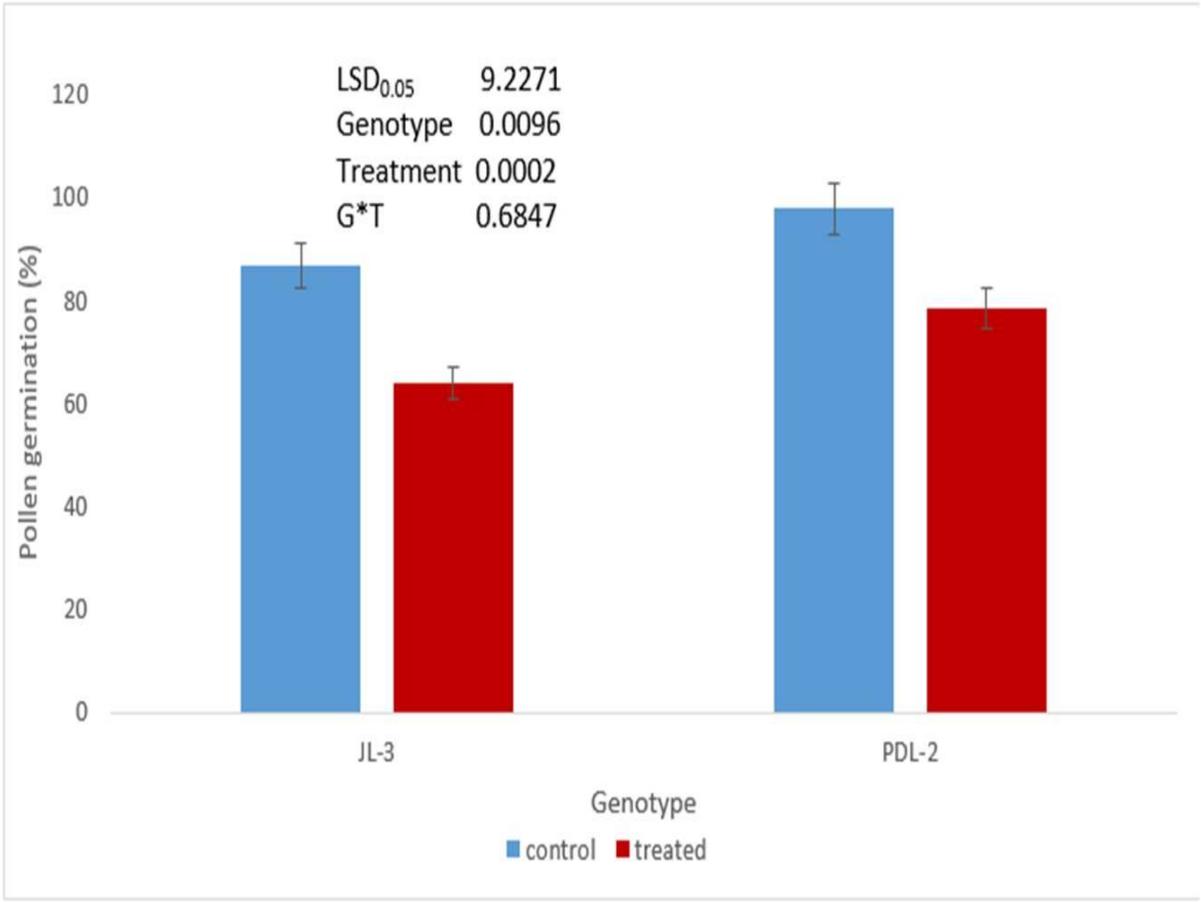

Genome wide transcriptome analysis reveals vital role of heat responsive genes in regulatory mechanisms of lentil (*Lens culinaris* Medikus)

Dharmendra Singh[1*], Chandan Kumar Singh[1], Jyoti Taunk[1], Vasudha Singh[1], Madan Pal[2*] & Kishor Gaikwad[3]

[1]Division of Genetics, ICAR-Indian Agricultural Research Institute, New Delhi-110012

[2]Division of Plant Physiology, ICAR-Indian Agricultural Research Institute, New Delhi-110012

[3]ICAR-National Research Centre on Plant Biotechnology, Pusa Campus, New Delhi-110012

*Corresponding authors emails: dharmendrapbg@rediffmail.com; madanpal@yahoo.com

Additional file 6. Fig S6 Stressed stigma (35^{0}C /20 ^{0}C) of sensitive genotype (JL-3) x stressed pollens (35^{0}C /20^{0}C) of tolerant genotype (PDL-2).

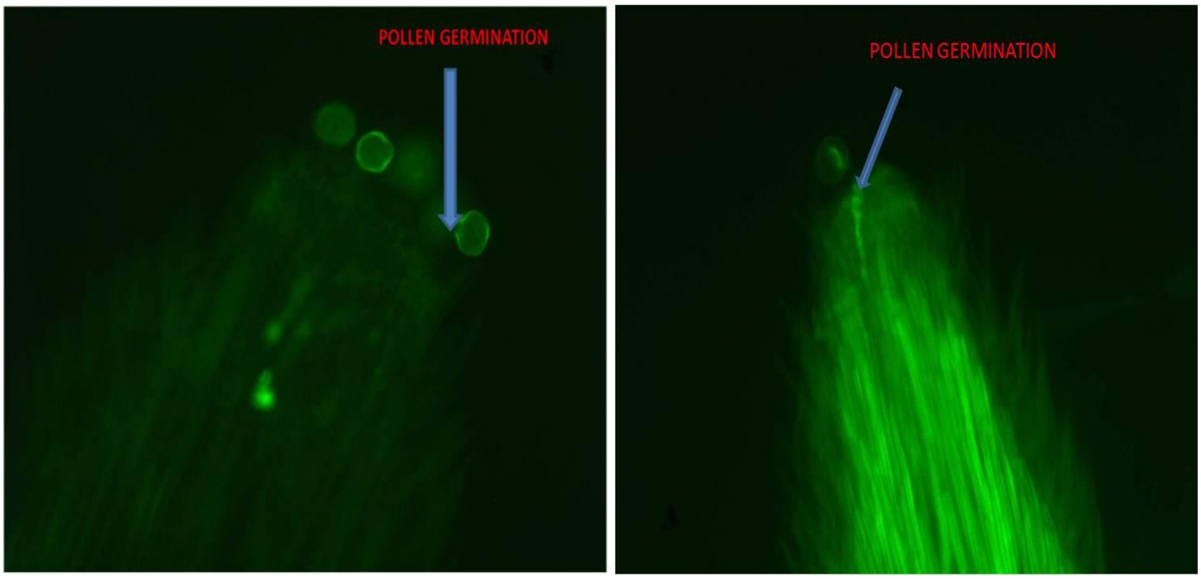

Genome wide transcriptome analysis reveals vital role of heat responsive genes in regulatory mechanisms of lentil (*Lens culinaris* Medikus)

Dharmendra Singh[1*], Chandan Kumar Singh[1], Jyoti Taunk[1], Vasudha Singh[1], Madan Pal[2*] & Kishor Gaikwad[3]

[1]Division of Genetics, ICAR-Indian Agricultural Research Institute, New Delhi-110012

[2]Division of Plant Physiology, ICAR-Indian Agricultural Research Institute, New Delhi-110012

[3]ICAR-National Research Centre on Plant Biotechnology, Pusa Campus, New Delhi-110012

*Corresponding authors emails: dharmendrapbg@rediffmail.com; madanpal@yahoo.com

Additional file 7. Fig S7. Stressed stigma of tolerant genotype (PDL-2) x stressed pollens (35^0C /20^0C) of sensitive genotype (JL-3).

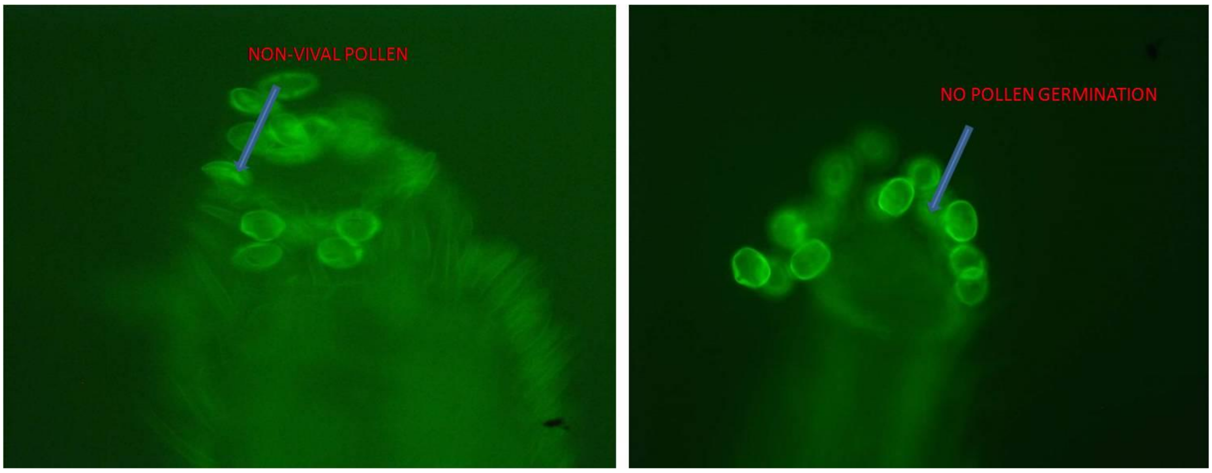

Genome wide transcriptome analysis reveals vital role of heat responsive genes in regulatory mechanisms of lentil (*Lens culinaris* Medikus)

Dharmendra Singh[1*], Chandan Kumar Singh[1], Jyoti Taunk[1], Vasudha Singh[1], Madan Pal[2*] & Kishor Gaikwad[3]

[1]Division of Genetics, ICAR-Indian Agricultural Research Institute, New Delhi-110012

[2]Division of Plant Physiology, ICAR-Indian Agricultural Research Institute, New Delhi-110012

[3]ICAR-National Research Centre on Plant Biotechnology, Pusa Campus, New Delhi-110012

*Corresponding authors emails: dharmendrapbg@rediffmail.com; madanpal@yahoo.com

Additional file 8. Fig S8 Principal component (PCA) analysis between the samples and their replicates. It depicts overall similarity between replicates whereas; variability between the samples under different conditions was observed.

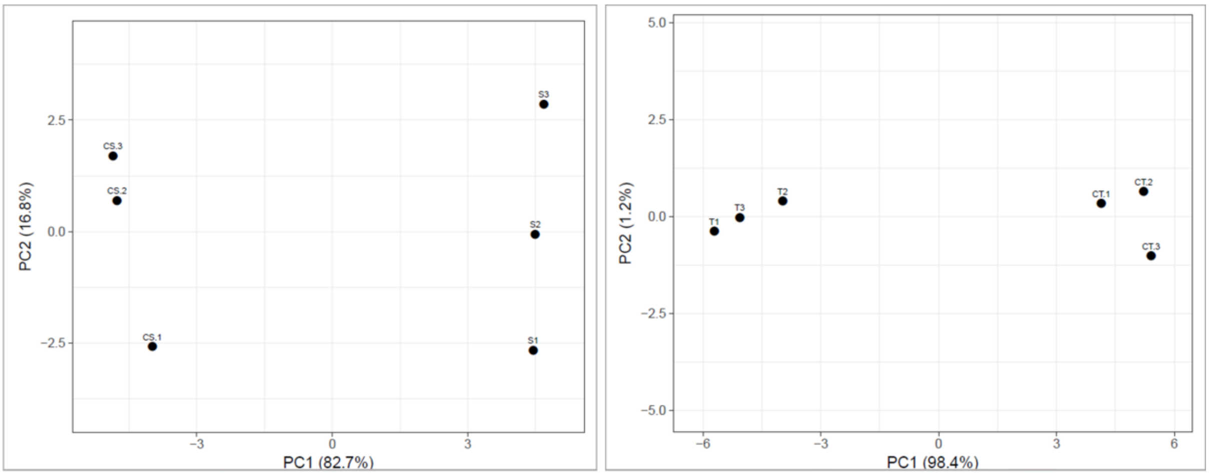

Control sensitive (CS) vs. Sensitive treated (S)   Control tolerant (CT) vs. Tolerant treated (T)

Genome wide transcriptome analysis reveals vital role of heat responsive genes in regulatory mechanisms of lentil (*Lens culinaris* Medikus)

Dharmendra Singh[1*], Chandan Kumar Singh[1], Jyoti Taunk[1], Vasudha Singh[1], Madan Pal[2*] & Kishor Gaikwad[3]

[1]Division of Genetics, ICAR-Indian Agricultural Research Institute, New Delhi-110012

[2]Division of Plant Physiology, ICAR-Indian Agricultural Research Institute, New Delhi-110012

[3]ICAR-National Research Centre on Plant Biotechnology, Pusa Campus, New Delhi-110012

*Corresponding authors emails: dharmendrapbg@rediffmail.com; madanpal@yahoo.com

Additional file 9: Fig. S9. HeatMap of top down regulated between samples with p value < 0.05 in Tolerant treated- Tolerant control.

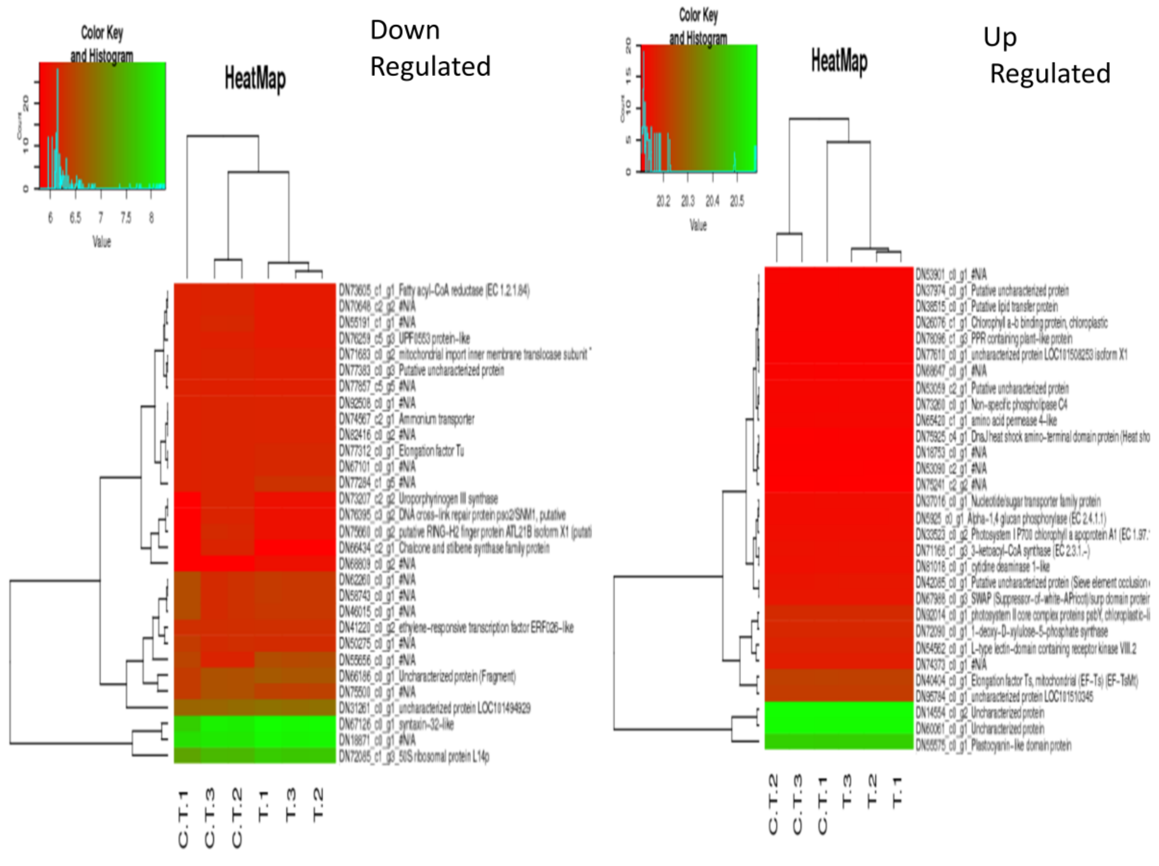

Genome wide transcriptome analysis reveals vital role of heat responsive genes in regulatory mechanisms of lentil (*Lens culinaris* Medikus)

Dharmendra Singh[1*], Chandan Kumar Singh[1], Jyoti Taunk[1], Vasudha Singh[1], Madan Pal[2*] & Kishor Gaikwad[3]

[1]Division of Genetics, ICAR-Indian Agricultural Research Institute, New Delhi-110012

[2]Division of Plant Physiology, ICAR-Indian Agricultural Research Institute, New Delhi-110012

[3]ICAR-National Research Centre on Plant Biotechnology, Pusa Campus, New Delhi-110012

*Corresponding authors emails: dharmendrapbg@rediffmail.com; madanpal@yahoo.com

Additional file 10: Fig. S10. HeatMap of top down and up regulated between samples with p value < 0.05 in sensitive treated- sensitive control.

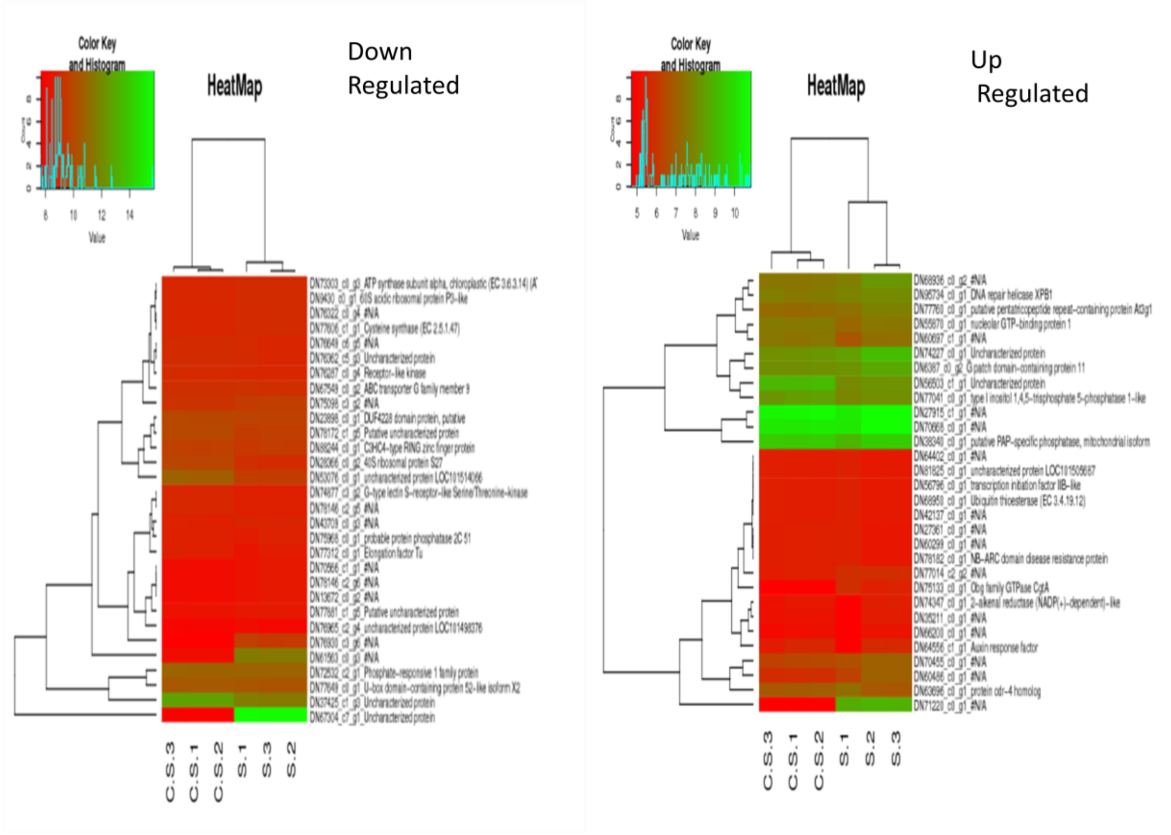

Genome wide transcriptome analysis reveals vital role of heat responsive genes in regulatory mechanisms of lentil (*Lens culinaris* Medikus)

Dharmendra Singh[1*], Chandan Kumar Singh[1], Jyoti Taunk[1], Vasudha Singh[1], Madan Pal[2*] & Kishor Gaikwad[3]

[1]Division of Genetics, ICAR-Indian Agricultural Research Institute, New Delhi-110012

[2]Division of Plant Physiology, ICAR-Indian Agricultural Research Institute, New Delhi-110012

[3]ICAR-National Research Centre on Plant Biotechnology, Pusa Campus, New Delhi-110012

*Corresponding authors emails: dharmendrapbg@rediffmail.com; madanpal@yahoo.com

Additional file 11: Fig. S11. Wego plot for GO terms for down regulated and up regulated transcripts in tolerant treated- tolerant control.

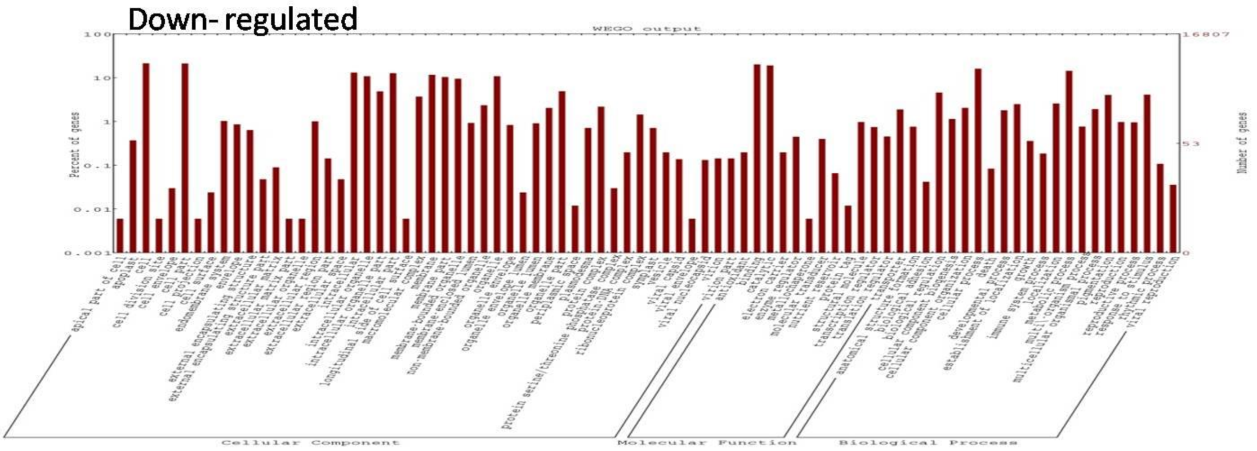

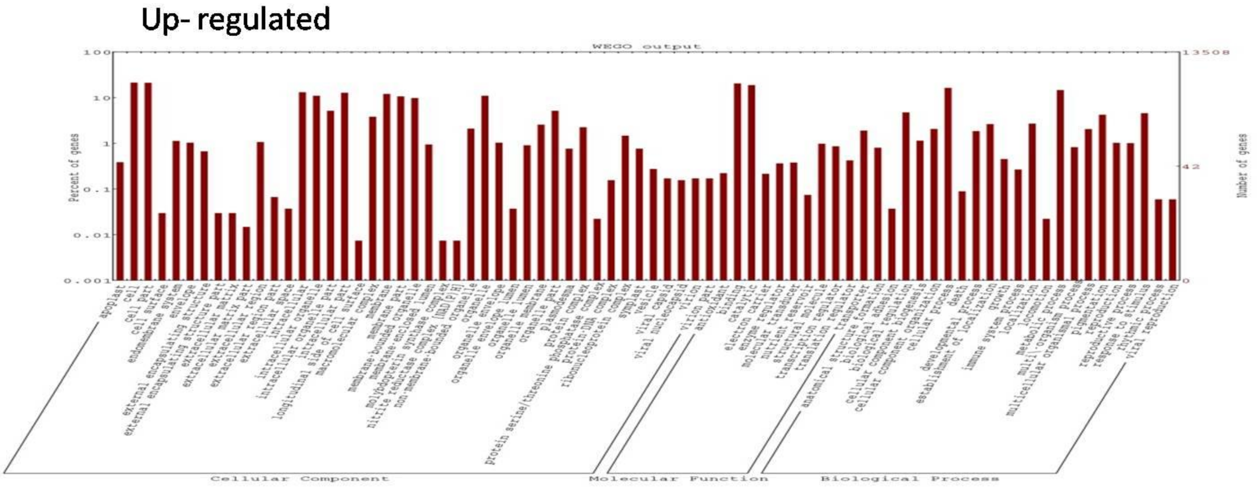

Genome wide transcriptome analysis reveals vital role of heat responsive genes in regulatory mechanisms of lentil (*Lens culinaris* Medikus)

Dharmendra Singh[1*], Chandan Kumar Singh[1], Jyoti Taunk[1], Vasudha Singh[1], Madan Pal[2*] & Kishor Gaikwad[3]

[1]Division of Genetics, ICAR-Indian Agricultural Research Institute, New Delhi-110012

[2]Division of Plant Physiology, ICAR-Indian Agricultural Research Institute, New Delhi-110012

[3]ICAR-National Research Centre on Plant Biotechnology, Pusa Campus, New Delhi-110012

*Corresponding authors emails: dharmendrapbg@rediffmail.com; madanpal@yahoo.com

Additional file 12: Fig. S12. Wego plot for GO terms for down regulated and up regulated transcripts in sensitive treated- sensitive control.

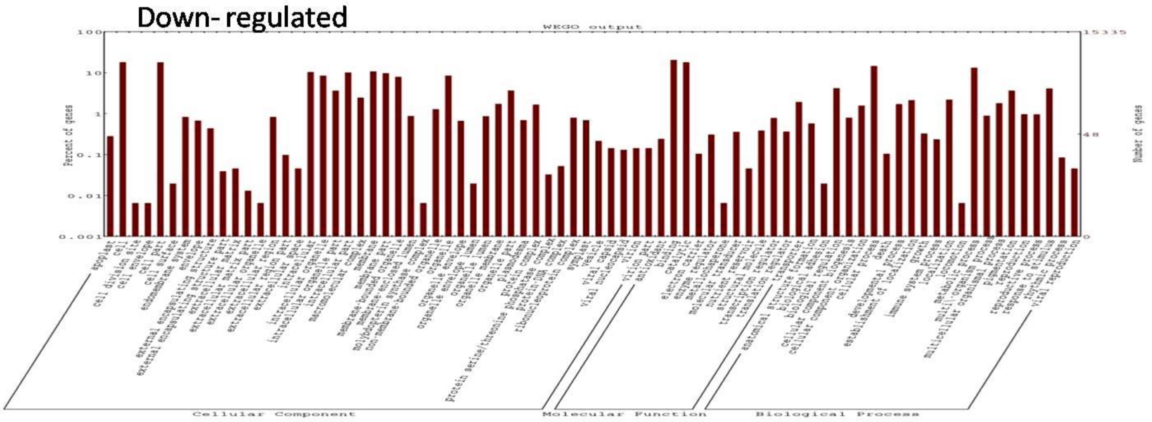

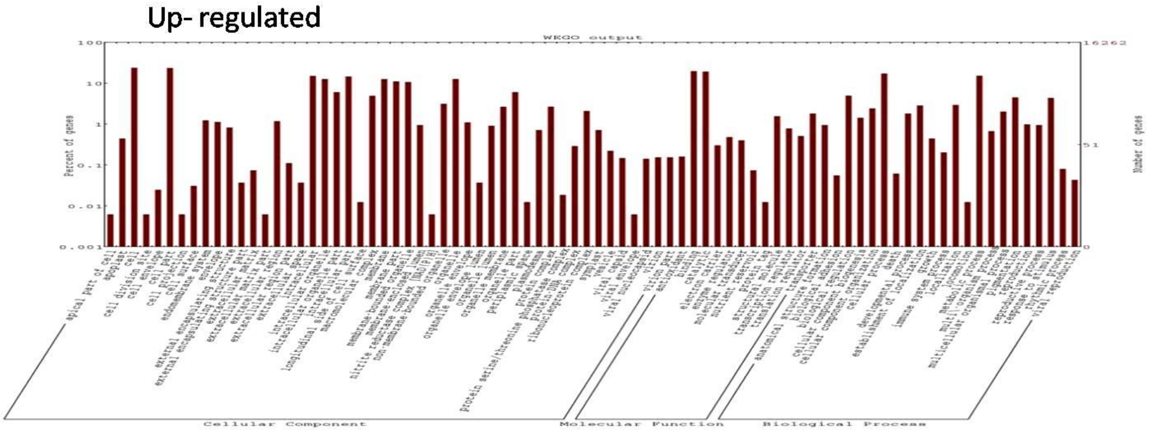

Genome wide transcriptome analysis reveals vital role of heat responsive genes in regulatory mechanisms of lentil (*Lens culinaris* Medikus)

Dharmendra Singh[1*], Chandan Kumar Singh[1], Jyoti Taunk[1], Vasudha Singh[1], Madan Pal[2*] & Kishor Gaikwad[3]

[1]Division of Genetics, ICAR-Indian Agricultural Research Institute, New Delhi-110012

[2]Division of Plant Physiology, ICAR-Indian Agricultural Research Institute, New Delhi-110012

[3]ICAR-National Research Centre on Plant Biotechnology, Pusa Campus, New Delhi-110012

*Corresponding authors emails: dharmendrapbg@rediffmail.com; madanpal@yahoo.com

Additional file 13: Fig. S13. MapMan display for tolerant treated- tolerant control, under heat stress. Up regulated genes are expressed in increased intense red while down regulated as blue at the amplitude of 4.5 to -4.5 (log2-value).

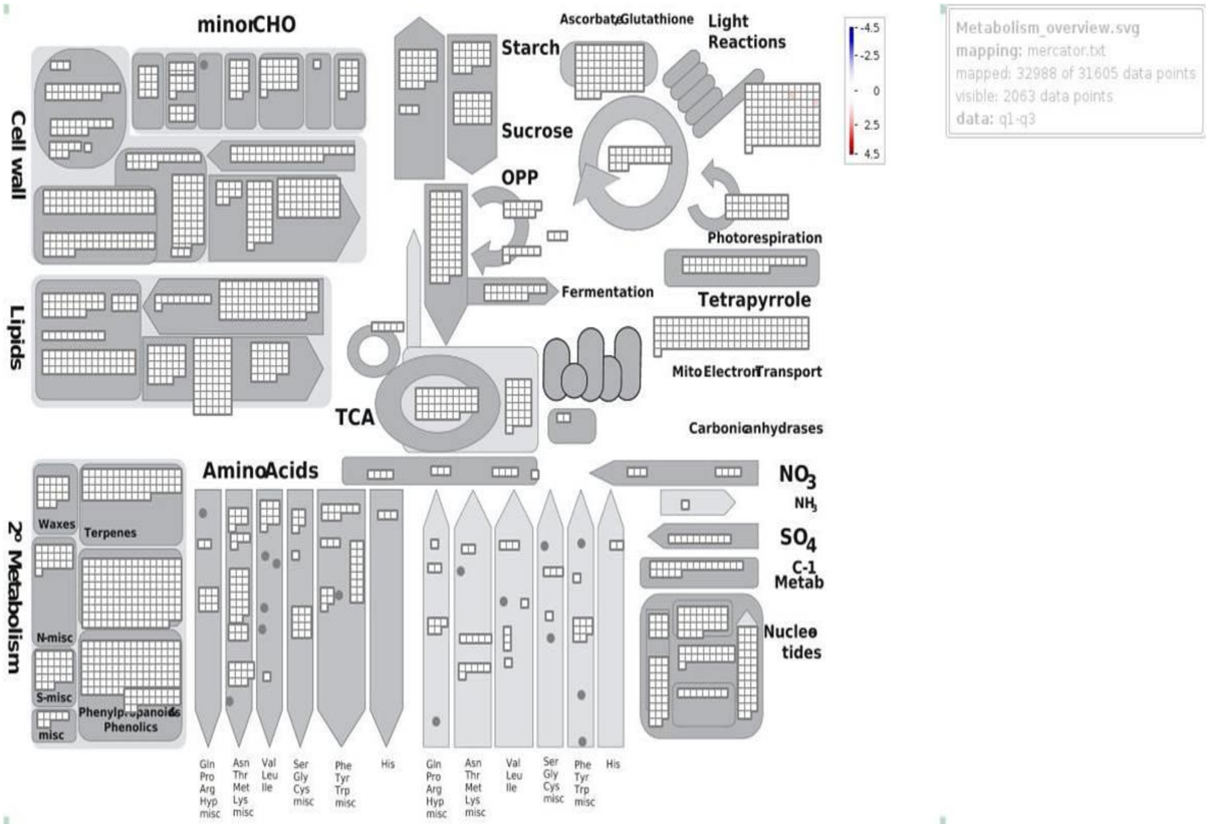

Genome wide transcriptome analysis reveals vital role of heat responsive genes in regulatory mechanisms of lentil (*Lens culinaris* Medikus)

Dharmendra Singh[1*], Chandan Kumar Singh[1], Jyoti Taunk[1], Vasudha Singh[1], Madan Pal[2*] & Kishor Gaikwad[3]

[1]Division of Genetics, ICAR-Indian Agricultural Research Institute, New Delhi-110012

[2]Division of Plant Physiology, ICAR-Indian Agricultural Research Institute, New Delhi-110012

[3]ICAR-National Research Centre on Plant Biotechnology, Pusa Campus, New Delhi-110012

*Corresponding authors emails: dharmendrapbg@rediffmail.com; madanpal@yahoo.com

Additional file 14: Fig. S14: MapMan display for sensitive treated- sensitive control, under heat stress. Up regulated genes are expressed in increased intense red while down regulated as blue at the amplitude of 4.5 to -4.5 (log2-value).

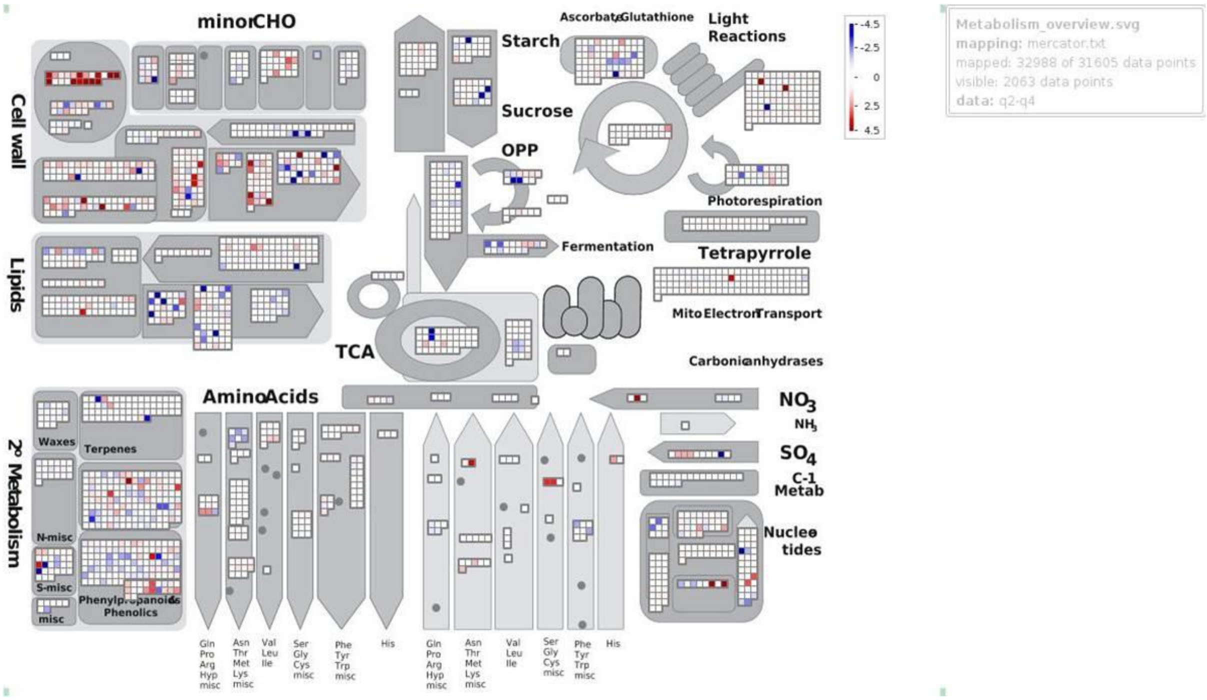

Genome wide transcriptome analysis reveals vital role of heat responsive genes in regulatory mechanisms of lentil (*Lens culinaris* Medikus)

Dharmendra Singh[1*], Chandan Kumar Singh[1], Jyoti Taunk[1], Vasudha Singh[1], Madan Pal[2*] & Kishor Gaikwad[3]

[1]Division of Genetics, ICAR-Indian Agricultural Research Institute, New Delhi-110012

[2]Division of Plant Physiology, ICAR-Indian Agricultural Research Institute, New Delhi-110012

[3]ICAR-National Research Centre on Plant Biotechnology, Pusa Campus, New Delhi-110012

*Corresponding authors emails: dharmendrapbg@rediffmail.com; madanpal@yahoo.com

Additional file 15: Fig. S15. Representative gel picture of Primer-10 (polymorphic SSR) on diverse genotypes.

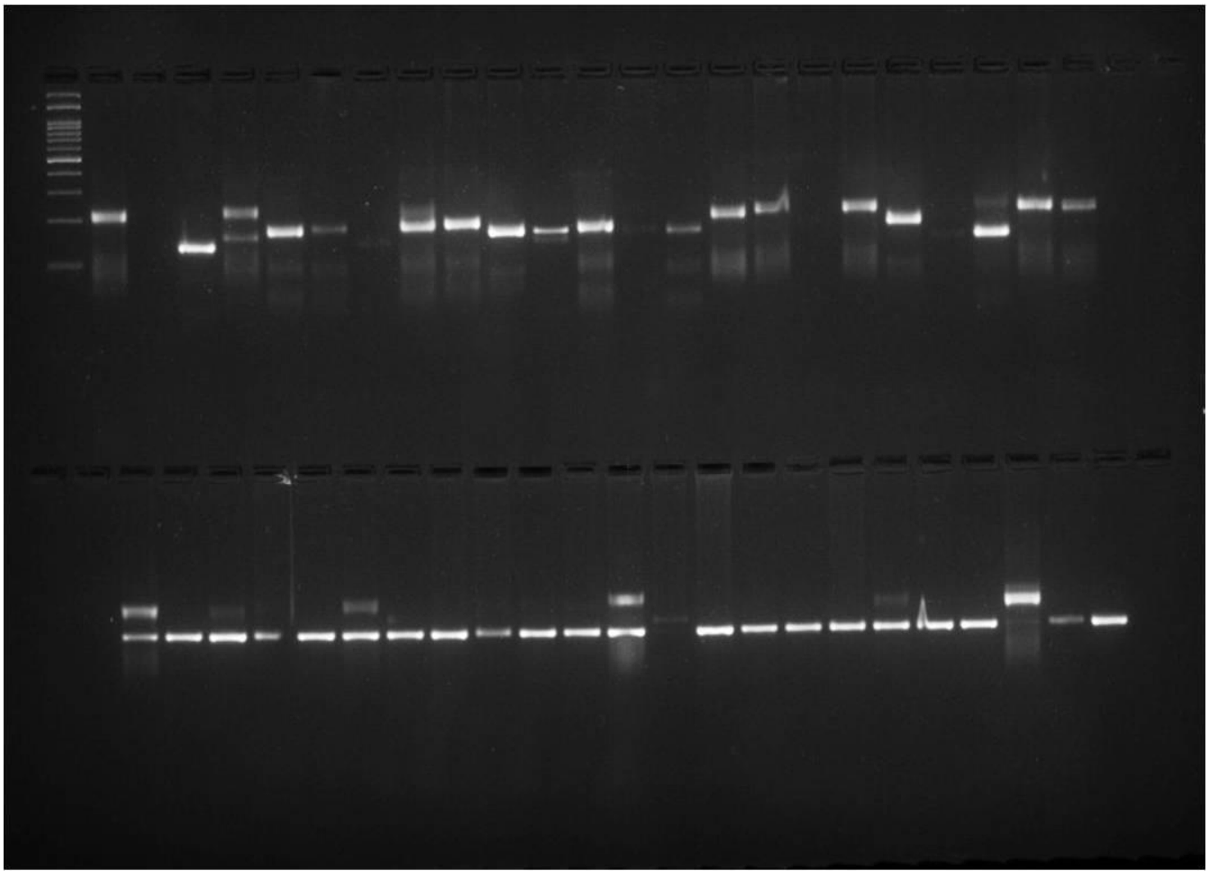

Additional File 16. Table S1. List of 96 genotypes used in validation of 55 g- SSRs.

S. No.	Genotype	Origin	Type	S. No.	Genotype	Origin	Type
1	BM-4	Bangladesh	Cult.	49	ILL-385	MEXICO	GC
2	DPL-62	India	Cult.	50	ILL-590	Turkey	GC
3	HUM-57	India	GC	51	ILL-7979	ICARDA	GC
4	FLIP-96-51	ICARDA	GC	52	ILL-7982	ICARDA	GC
5	IG-109039	ICARDA	GC	53	ILL-91887	ICARDA	GC
6	IG-111991	ICARDA	LR	54	ILL-9841	ICARDA	GC
7	IG-112078	ICARDA	LR	55	ILL-9900	ICARDA	GC
8	IG-11210	ICARDA	LR	56	ILL-9916	ICARDA	GC
9	IG-112128	ICARDA	LR	57	ILL-9941	ICARDA	GC
10	IG-112131	ICARDA	LR	58	ILL-9960	ICARDA	GC
11	IG-112137	ICARDA	LR	59	ILWL-06	Turkey	Wild
12	IG-116551	ICARDA	LR	60	ILWL-100	Turkey	Wild
13	IG-129185	ICARDA	LR	61	ILWL-142	Syria	Wild
14	IG-129214	ICARDA	LR	62	ILWL-165	Syria	Wild
15	IG-129287	ICARDA	LR	63	ILWL-20	Palestine	Wild
16	IG-129309	ICARDA	LR	64	ILWL-203	Turkey	Wild
17	IG-129315	ICARDA	LR	65	ILWL-23	Italy	Wild
18	IG-12970	ICARDA	LR	66	ILWL-29	Spain	Wild
19	IG-130219	ICARDA	LR	67	ILWL-314	Turkey	Wild
20	IG-130272	ICARDA	LR	68	ILWL-340	Jordan	Wild
21	IG-136607	ICARDA	LR	69	ILWL-350	Syria	Wild

22	IG-136608	-	Wild	70	ILWL-357	Syria	Wild
23	IG-136612	Turkey	Wild	71	ILWL-398(A)	Lebanon	Wild
24	IG-136637	France	Wild	72	ILWL-415	Syria	Wild
25	IG-136653	Israel	Wild	73	ILWL-418	Syria	Wild
26	IG-149	ICARDA	LR	74	ILWL-437	Turkey	Wild
27	IG-69549	ICARDA	LR	75	ILWL-447	Turkey	Wild
28	IG-71630	ICARDA	LR	76	ILWL-55(2)	Israel	Wild
29	IG-73717	ICARDA	LR	77	ILWL-468	-	wild
30	IG-73802	ICARDA	LR	78	IPL-406	India	Cult.
31	IG-73945	ICARDA	LR	79	JL-3	India	Cult.
32	IG-75920	ICARDA	LR	80	L-4602	India	BL
33	ILL-10031	ICARDA	GC	81	L-7903	India	BL
34	ILL-10032	ICARDA	GC	82	LC-282-896	India	BL
35	ILL-10040	ICARDA	GC	83	LC-284-116	India	BL
36	ILL-10063	ICARDA	GC	84	LC-289-1447	India	BL
37	ILL-10074	ICARDA	GC	85	LC-292-1544	India	BL
38	ILL-10075	ICARDA	GC	86	LC-292-997	India	BL
39	ILL-10082	ICARDA	GC	87	LC-300-11	India	BL
40	ILL-10133	ICARDA	GC	88	LC-300-12	India	BL
41	ILL-10234	ICARDA	GC	89	LC-300-13	India	BL
42	ILL-10266	ICARDA	GC	90	LC-300-8	India	BL
43	ILL-10270	ICARDA	GC	91	LC-300-9	India	BL
44	ILL-10671	-	GC	92	LC-74-1-51	India	BL
45	ILL-10809	ICARDA	GC	93	PDL-1	ICARDA	BL
46	ILL-10826	ICARDA	GC	94	PDL-2	ICARDA	BL
47	ILL-10897	ICARDA	GC	95	PSL-9	India	BL
48	ILL-10951	ICARDA	GC	96	WBL-77	India	Cult.

Germplasm collection = GC, Breeding Line = BL, Cultivar = Cult., Landrace = LR,

Additional File 17. Table 2: List of 55 g-SSRs developed for validation in 96 genotypes.

Primer No.	Seq ID	Orientation	tm	GC%	Seq
Primer-1	TRINITY_DN52494_c0_g1_i1	FORWARD	54.84	38.1	TGGACTTTAAGCATGATAAGC
	TRINITY_DN52494_c0_g1_i1	REVERSE	54.94	38.1	ACTTTGCCTTCCCTTCTATTA
Primer-2	TRINITY_DN17284_c2_g2_i1	FORWARD	54.95	47.62	GGCATTCTCAGTCACATAGAG
	TRINITY_DN17284_c2_g2_i1	REVERSE	55.27	38.1	TGTTATCATATGCCGATCTTC
Primer-3	TRINITY_DN17275_c0_g1_i1	FORWARD	55.24	38.1	CTAGACAATGTCCAAAATCCA
	TRINITY_DN17275_c0_g1_i1	REVERSE	55.06	33.33	AATTGTGACAAATCATCAAGG
Primer-4	TRINITY_DN52494_c0_g1_i1	FORWARD	54.89	33.33	TCCTTTCTTTTGATGACCATA
	TRINITY_DN52494_c0_g1_i1	REVERSE	54.62	38.1	TATGGTTTCTGATGCTTCTTC
Primer-5	TRINITY_DN52424_c0_g1_i1	FORWARD	55.51	47.62	GGACGGACTTTTGTATAGGAG
	TRINITY_DN52424_c0_g1_i1	REVERSE	55.12	47.62	ATAGTTGGATCCTGCTCTCTC
Primer-6	TRINITY_DN17275_c1_g1_i5	FORWARD	54.97	42.86	CTTTCACCAGTCTAATCATGC
	TRINITY_DN17275_c1_g1_i5	REVERSE	55.28	38.1	TAGAAAGATTGTGGCAGAAGA
Primer-7	TRINITY_DN17285_c1_g11_i1	FORWARD	55.93	42.86	CTTTGACGATACTGACGAATG
	TRINITY_DN17285_c1_g11_i1	REVERSE	55.02	38.1	TCATACAGCTCTTTTTCTTGC
Primer-8	TRINITY_DN17266_c1_g4_i1	FORWARD	55.13	33.33	CTTTTTCCTCAATCCTTCATT
	TRINITY_DN17266_c1_g4_i1	REVERSE	55.26	47.62	GAGAGGGATCTACGAGAAGAA
Primer-9	TRINITY_DN17294_c2_g1_i1	FORWARD	55.41	38.1	AACAATGTTGGTGACACACTT
	TRINITY_DN17294_c2_g1_i1	REVERSE	54.58	38.1	CATTATTTCCTGACTGCTGTT
Primer-10	TRINITY_DN52428_c0_g1_i1	FORWARD	54.91	38.1	ATTATCCCTTATCCGATCTTG
	TRINITY_DN52428_c0_g1_i1	REVERSE	54.5	38.1	AACGTTGGTTAGTTCAGAATG
Primer-11	TRINITY_DN17236_c3_g1_i1	FORWARD	55.44	38.1	TCCTCTTTTTGATAAGCCTTC
	TRINITY_DN17236_c3_g1_i1	REVERSE	54.83	42.86	TACCCCAACCACAATATACAC
Primer-12	TRINITY_DN17233_c0_g1_i5	FORWARD	55.81	33.33	ACGACAACAAAATCAGTTGAA
	TRINITY_DN17233_c0_g1_i5	REVERSE	54.67	33.33	CAATAATAAAAGTGCGGTGTT
Primer-13	TRINITY_DN52421_c0_g1_i1	FORWARD	54.89	42.86	AAGAAGGTGAAGAGAAGGAGA
	TRINITY_DN52421_c0_g1_i1	REVERSE	55.38	47.37	AAACTCCTCACCACGATGT
Primer-14	TRINITY_DN17222_c1_g2_i4	FORWARD	55.57	33.33	ACTAAATGGTGCTTTGGTTTT
	TRINITY_DN17222_c1_g2_i4	REVERSE	55.16	38.1	TCCTTGGACATAAAAGCTACA
Primer-15	TRINITY_DN52408_c0_g1_i1	FORWARD	55.1	47.62	CAAGTAGCTAGTGATGGTTGG
	TRINITY_DN52408_c0_g1_i1	REVERSE	54.33	33.33	TGAAAAACACTATTCCCATTC
Primer-16	TRINITY_DN52473_c0_g1_i1	FORWARD	55.15	40	ATGCTCGTGCAAAGACTATT
	TRINITY_DN52473_c0_g1_i1	REVERSE	54.94	38.1	AGAGAGAAGGCAAATGAAGTT
Primer-17	TRINITY_DN17236_c0_g1_i2	FORWARD	55.39	38.1	TGCAGGAAATGTAGAATTGTC
	TRINITY_DN17236_c0_g1_i2	REVERSE	55.29	52.38	CTGCACTTCCTACAGTGAGAG
Primer-18	TRINITY_DN17204_c0_g1_i1	FORWARD	55.56	42.86	GACAAATTCATCTGGAGTGTG
	TRINITY_DN17204_c0_g1_i1	REVERSE	54.89	38.1	GGATATCATGGAGATGAACAA
Primer-19	TRINITY_DN17266_c4_g3_i1	FORWARD	54.82	42.86	CTGACCCATTAAGATGTGAAG
	TRINITY_DN17266_c4_g3_i1	REVERSE	59.97	55.56	GCTTCATCAGCAGCAGCA
Primer-20	TRINITY_DN17294_c2_g3_i3	FORWARD	54.7	38.1	TATGTTTAATGCTTCCCAGAG
	TRINITY_DN17294_c2_g3_i3	REVERSE	53.88	38.1	GAGATTGCATCTGAAGCTTAT
Primer-21	TRINITY_DN52456_c0_g1_i1	FORWARD	55.15	38.1	AAAGAACCCAGCTCATAAATC
	TRINITY_DN52456_c0_g1_i1	REVERSE	55.16	33.33	AGCAAGAAAGGCTTGTTATTT

Primer-22	TRINITY_DN52455_c0_g1_i1	FORWARD	55.32	42.86	CTATCCAAGATCCAATCCTTC
	TRINITY_DN52455_c0_g1_i1	REVERSE	55.05	50	CAGAAGAGATGGAAGACTGC
Primer-23	TRINITY_DN17244_c0_g1_i1	FORWARD	55.46	38.1	CAAAGACTGTGTTTTGTTCGT
	TRINITY_DN17244_c0_g1_i1	REVERSE	54.99	42.86	CTGAGCCGAAAAACTAGTACA
Primer-24	TRINITY_DN17236_c0_g1_i4	FORWARD	54.73	31.82	CCTTTCTATATGCAAACAATCA
	TRINITY_DN17236_c0_g1_i4	REVERSE	54.63	38.1	CAACACACATGTCTTCTTTGA
Primer-25	TRINITY_DN17204_c0_g1_i7	FORWARD	55.36	38.1	TCGGATCCTAAAAGATACCAT
	TRINITY_DN17204_c0_g1_i7	REVERSE	53.49	38.1	CAAGGAAGTTCCATATTGAGT
Primer-26	TRINITY_DN17238_c1_g1_i2	FORWARD	55.09	33.33	GTTTCGATTTTGTTAGGGTTT
	TRINITY_DN17238_c1_g1_i2	REVERSE	55.33	42.86	ATAGCTCACTGTTCGCAGATA
Primer-27	TRINITY_DN17210_c0_g3_i1	FORWARD	54.38	38.1	TTCTTGTTGGATCTCTCATTC
	TRINITY_DN17210_c0_g3_i1	REVERSE	55.14	42.86	GGTTTACCGAGTCAGAAGTTT
Primer-28	TRINITY_DN17222_c1_g3_i3	FORWARD	55.01	38.1	AATGGAAATTGGAGTAAGAGG
	TRINITY_DN17222_c1_g3_i3	REVERSE	54.93	42.86	AACACTCCAGGCATTGTAGTA
Primer-29	TRINITY_DN52476_c0_g1_i1	FORWARD	55.52	45	ACGACTTCAGTTGAAACAGC
	TRINITY_DN52476_c0_g1_i1	REVERSE	55.31	42.86	TCTTGAGATTTGTAGGTGTGG
Primer-30	TRINITY_DN17296_c0_g1_i1	FORWARD	55.81	36.36	GCCATGCCATCATAGTTATATT
	TRINITY_DN17296_c0_g1_i1	REVERSE	54.86	33.33	AGGAATGGAGAAAGAAGAAAA
Primer-31	TRINITY_DN17216_c0_g1_i2	FORWARD	55	42.86	ATGGTGTTGGTAGAGTCAATG
	TRINITY_DN17216_c0_g1_i2	REVERSE	55.63	38.1	ATGAAGATCTTTTCCACCATC
Primer-32	TRINITY_DN17217_c0_g2_i2	FORWARD	55.12	38.1	CCTAATGCAATTAAGTCATGC
	TRINITY_DN17217_c0_g2_i2	REVERSE	54.39	42.86	CACTGTACTGATGTCATGGAA
Primer-33	TRINITY_DN17204_c0_g1_i9	FORWARD	54.95	31.82	TTGACAATGAGAAAGTGTGTTT
	TRINITY_DN17204_c0_g1_i9	REVERSE	55.1	42.86	GTCGGTGTCCTATATTTGTGA
Primer-34	TRINITY_DN17238_c2_g3_i1	FORWARD	54.69	33.33	GAATTATTTAACCGCGTATGA
	TRINITY_DN17238_c2_g3_i1	REVERSE	53.89	33.33	AACATTTACCGTTAAAACTCG
Primer-35	TRINITY_DN17294_c2_g3_i3	FORWARD	55.59	38.1	CAGCACAATCAGCAATCTAAT
	TRINITY_DN17294_c2_g3_i3	REVERSE	55.26	38.1	ATATTGACTGTTGAGCCATTG
Primer-36	TRINITY_DN17218_c0_g1_i1	FORWARD	55.1	33.33	TAGATTGCTTTGTGGGAATAA
	TRINITY_DN17218_c0_g1_i1	REVERSE	55.04	33.33	AGAAAGCTTTTCCTTGTTTGT
Primer-37	TRINITY_DN17282_c1_g5_i1	FORWARD	55.42	42.86	GAGGAAGAATTCAGTTTGGAC
	TRINITY_DN17282_c1_g5_i1	REVERSE	54.94	38.1	TGCACACAAATGTTAGTTACG
Primer-38	TRINITY_DN17242_c2_g1_i2	FORWARD	55.02	33.33	TATTGAAGCTGGAATCCATAA
	TRINITY_DN17242_c2_g1_i2	REVERSE	52.03	42.86	TACTCAAGGTCTGAACATAGG
Primer-39	TRINITY_DN52433_c0_g1_i1	FORWARD	55.03	38.1	ACTTTCTCAACAATCCCTTTC
	TRINITY_DN52433_c0_g1_i1	REVERSE	55.32	33.33	GCCAATCATAAAAGACAATCA
Primer-40	TRINITY_DN17296_c0_g1_i1	FORWARD	54.72	38.1	CAATATTGTGCCTATTCATCC
	TRINITY_DN17296_c0_g1_i1	REVERSE	55.03	38.1	GCAACATAGCATTTGGTAAAC
Primer-	TRINITY_DN17296_c0_g1_i1	FORWARD	55.39	38.1	TGCAGGAAATGTAGAATTGTC

41					
	TRINITY_DN17296_c0_g1_i1	REVERSE	55.29	52.38	CTGCACTTCCTACAGTGAGAG
Primer-42	TRINITY_DN17272_c0_g4_i1	FORWARD	55.13	38.1	ACTCCAACACAAGATCAAATG
	TRINITY_DN17272_c0_g4_i1	REVERSE	55.07	42.86	ATACTGGAAAGAAAGGTCCAG
Primer-43	TRINITY_DN17239_c0_g2_i7	FORWARD	54.2	38.1	AAACATTAGCAGGTACTTGGA
	TRINITY_DN17239_c0_g2_i7	REVERSE	55.05	42.86	CAGTTAGTTGAAGATGCCAAC
Primer-44	TRINITY_DN17238_c2_g5_i1	FORWARD	54.79	42.86	GTAAATGTGTTCCCGTACAAG
	TRINITY_DN17238_c2_g5_i1	REVERSE	54.74	38.1	ATGGTTCAAGAGATGAGACAA
Primer-45	TRINITY_DN17218_c0_g1_i1	FORWARD	55	38.1	GAAGAAGATGATGATGATGGA
	TRINITY_DN17218_c0_g1_i1	REVERSE	55.04	33.33	AGAAAGCTTTTCCTTGTTTGT
Primer-46	TRINITY_DN17204_c0_g2_i1	FORWARD	54.67	38.1	CGTGTATGATGATGAATAGCA
	TRINITY_DN17204_c0_g2_i1	REVERSE	55.18	33.33	TTTTGCAGTCAAACTTCTCAT
Primer-47	TRINITY_DN17228_c0_g1_i3	FORWARD	54.21	42.86	CTTCCTACGAATACCGTAATG
	TRINITY_DN17228_c0_g1_i3	REVERSE	54.49	42.86	TCCTGATTGCTACACTATTCC
Primer-48	TRINITY_DN17281_c0_g1_i7	FORWARD	55.36	36.36	GCTCAGTATAGATGGCAATTTT
	TRINITY_DN17281_c0_g1_i7	REVERSE	54.2	42.86	CAATACAAGTCCAGAGATGGT
Primer-49	TRINITY_DN17251_c0_g1_i1	FORWARD	54.7	38.1	CATTTCCCAGCTAAACATCTA
	TRINITY_DN17251_c0_g1_i1	REVERSE	54.76	38.1	GGGAAGATATCAAAGTCCATT
Primer-50	TRINITY_DN17217_c0_g1_i5	FORWARD	55.05	38.1	TCAAACAAAGCCTCAGTGTAT
	TRINITY_DN17217_c0_g1_i5	REVERSE	54.33	33.33	TGTAAAGAACATTCCATTTCC
Primer-51	TRINITY_DN17259_c3_g1_i13	FORWARD	54.92	38.1	ACTGACTGAACAGTTTTTCCA
	TRINITY_DN17259_c3_g1_i13	REVERSE	55.04	33.33	GAGGGTTTGAATTTTCTTGTT
Primer-52	TRINITY_DN17272_c1_g1_i1	FORWARD	55.3	38.1	ATGATCTCCTTTGGAAACTGT
	TRINITY_DN17272_c1_g1_i1	REVERSE	55.15	38.1	GGCTTAGATCCATCTTTTGTT
Primer-53	TRINITY_DN17210_c0_g3_i1	FORWARD	54.62	42.86	GGACTAACGATGACAATGTTC
	TRINITY_DN17210_c0_g3_i1	REVERSE	55.2	33.33	ACATCGAATCCATTTTCCTAT
Primer-54	TRINITY_DN17207_c0_g2_i18	FORWARD	54.58	33.33	TGATTCAATCCCAGTGTATTT
	TRINITY_DN17207_c0_g2_i18	REVERSE	54.38	38.1	TCTTTCACCATCTCATCTTTC
Primer-55	TRINITY_DN52449_c0_g1_i1	FORWARD	54.94	38.1	AGAGAAGAAGCCATTTTCAGT
	TRINITY_DN52449_c0_g1_i1	REVERSE	54.89	33.33	ATACAAGGTAACATTGCCAAA

Polymorphic markers are highlighted.

Additional file 18. Table S3. Shortlisted genes with their primer sequences for validation though RT-PCR in tolerant vs sensitive combinations based on Log FC and FDR

S. No.	ID	Gene name	Primer name	Primer sequence
1	TRINITY_DN68508_c0_g1	MTR_7g092380 MtrDRAFT_AC155881g12v1	MTR_7g092380_F	TCGCTTTGATTGCTTTGATG
			MTR_7g092380_R	CATATCACAACGCCGAAATG
2	TRINITY_DN32858_c0_g1	LOC101499292	LOC101499292_F	TTCTCTCCAACACGGAGCTT
			LOC101499292_R	AGTCCAGGCATATCCAAACG
3	TRINITY_DN74582_c1_g3	MTR_4g114950	MTR_4g114950_F	GAACACGTGGCTTGATGAGA
			MTR_4g114950_R	GGGGTCAAGAATGGATCAGA
4	TRINITY_DN71099_c0_g1	PHAVU_001G039700g	PHAVU_001G039700g_F	GCACCAACTGATGGACCTTT
			PHAVU_001G039700g_R	CGGCATATCGATCACAAACA
5	TRINITY_DN60178_c0_g1	MTR_2g014470 MtrDRAFT_AC158497g3v2	MTR_2g014470_F	TGCCTTCTTTTGGAACAACC
			MTR_2g014470_R	GGAAACGAGCAACCAAATGT
6	TRINITY_DN68353_c0_g2	MTR_3g086050	MTR_3g086050_F	CTCAGAGCCATGACAGGTGA
			MTR_3g086050_R	TGCAAATGGATCTTCCATCA
7	TRINITY_DN29122_c0_g2	MTR_1g062190	MTR_1g062190_F	ACCACTGGTTCGGTGCTTAC
			MTR_1g062190_R	AGCTCATCGGGAACTTGAGA
8	TRINITY_DN10549_c0_g1	MTR_5g078040	MTR_5g078040_F	GCCAAACAATCTCCTTTCCA
			MTR_5g078040_R	TGGGCAATCCTCCAATTTAC
9	TRINITY_DN5198_c0_g1	UGT73AC1	UGT73AC1_F	TGTGCTGGTGTGCCTATGAT
			UGT73AC1_R	CTGCAAACTCCACCTTCTCC
10	TRINITY_DN54817_c0_g1	LOC101501134	LOC101501134_F	TCGGTATGGTGAGGAAAGG
			LOC101501134_R	AGCACCGCCACTAACTCAAT

Additional file 19. Table S4. Total Input reads, read mapped and mapping rate of transcriptome assembly.

Properties	Input Reads	Filtered Reads	Reads Mapped	Overall Read Mapping Rate	Aligned Pairs	Concordant Pair Alignment Rate
Tolerant-Treated_1	22536460	22530924	19719402	87.52%	21064767	82.90%
Tolerant-Treated _2	24355081	24354870	21310695	87.50%	20241302	82.90%
Tolerant-Treated_3	23604075	23600914	20653565	87.51%	19623949	82.90%
Sensitive-Treated_1	26210135	26209794	22409665	85.50%	21249126	80.90%
Sensitive-Treated_2	22541528	22538749	19273006	85.51%	20430391	80.90%
Sensitive-Treated_3	23960480	23957250	20486210	85.51%	19404026	80.80%
Tolerant-Control_1	26685338	26683165	23322985	87.41%	22118262	82.70%
Tolerant-Control_2	24935410	24933624	21818483	87.51%	20719399	82.90%
Tolerant-Control_3	28521319	28520206	24899111	87.30%	21486289	82.60%
Sensitive-Control_1	30210135	30208348	26313027	87.11%	25001244	82.50%
Sensitive-Control_2	27960145	27968244	24381246	87.17%	23163895	82.60%
Sensitive-Control_3	32460168	32454893	28078045	86.51%	26462235	81.30%

Additional figure 20. Table S5. Total number of SSRs, SNPs and InDels generated from different combinations

Properties	Total number of reads	Total number of SSRs	Total number of SNPs	Total number of InDels
Tolerant- Treated_1	25355081	10474	17637	645
Tolerant- Treated _2	24355081	10214	16941	615
Tolerant- Treated _3	23605081	10013	16752	606
Sensitive-Treated_1	26210135	11275	17842	683
Sensitive-Treated_2	25210135	10928	17218	636
Sensitive-Treated_3	23960135	10678	16442	597
Tolerant-Control_1	26685338	11236	17475	663
Tolerant-Control_2	24935338	10348	17076	633
Tolerant-Control_3	25935338	10925	17120	627
Sensitive-Control_1	30210135	19167	13306	574
Sensitive-Control_2	27960145	11929	12715	552
Sensitive-Control_3	32460145	13863	13654	557

Additional file 21. Table S6: Major allelic frequency, number of alleles, genetic diversity, PIC and heterozygosity of 18 polymorphic g-SSRs

Marker	Major.Allele.Frquency	AlleleNo	GeneDiversity	Heterozygosity	PIC
**p_13**	0.8854	3.0000	0.2081	0.0000	0.1957
**p_15**	0.8333	3.0000	0.2908	0.0000	0.2702
**P_18**	0.7396	3.0000	0.4178	0.0000	0.3786
**p_4**	0.8542	3.0000	0.2598	0.0000	0.2442
**p_8**	0.5521	3.0000	0.5662	0.0000	0.4850
**P_31**	0.9271	3.0000	0.1365	0.0000	0.1296
**p_39**	0.6771	5.0000	0.5048	0.0000	0.4705
**p_40**	0.6146	4.0000	0.5276	0.0208	0.4554
**p_3**	0.9167	3.0000	0.1554	0.0000	0.1481
**p_44**	0.8021	3.0000	0.3305	0.0000	0.2968
**p_27**	0.9063	3.0000	0.1743	0.0000	0.1669
**P_10**	0.2917	10.0000	0.8461	0.1042	0.8304
**P_11**	0.7083	4.0000	0.4568	0.0000	0.4148
**P_29**	0.5625	6.0000	0.6357	0.1250	0.6039
**P_30**	0.8438	5.0000	0.2787	0.0313	0.2653
**p_26**	0.8073	4.0000	0.3215	0.0208	0.2866
**p_53**	0.6406	7.0000	0.5652	0.0208	0.5447
**p_55**	0.6042	5.0000	0.5653	0.0000	0.5124
**p_28**	0.7083	4.0000	0.4464	0.0000	0.3940
**Mean**	0.7303	4.2632	0.4046	0.0170	0.3733

Additional file 22. Excel sheet: EdgeR data file containing all the transcripts, annotations and their corresponding fold change values for the three combinations.
